# Graph designs for deep learning–based multi-omics integration

**DOI:** 10.1093/bib/bbag410

**Published:** 2026-07-31

**Authors:** Muhtasim Noor Alif, Khandakar Tanvir Ahmed, Sudipto Baul, Wei Zhang

**Affiliations:** Department of Computer Science, University of Central Florida, 4000 Central Florida Blvd., Orlando, FL 32816, United States; Department of Computer Science, University of Central Florida, 4000 Central Florida Blvd., Orlando, FL 32816, United States; Department of Computer Science, University of Central Florida, 4000 Central Florida Blvd., Orlando, FL 32816, United States; Department of Computer Science, University of Central Florida, 4000 Central Florida Blvd., Orlando, FL 32816, United States

**Keywords:** multi-omics, node schema, edge semantics, integration strategy, graph context

## Abstract

Modern sequencing technologies can now capture multiple omic layers from the same biological system, but integrating these views into a coherent model is far from trivial. Graph-based deep learning has become an attractive strategy because it can represent complex molecular interactions and sample relationships in a flexible way. In this review, we survey how graphs are constructed and used in multi-omics deep learning models, organizing methods by node schema, edge semantics, interaction type, integration strategy, graph context, and model architecture across bulk, single-cell, and spatial settings. We summarize the strengths and weaknesses of different design choices in terms of interpretability, data requirements, robustness to noise and missing modalities, and suitability for tasks ranging from prediction to mechanism-oriented discovery. Based on these insights, we outline a general, practical pipeline for constructing, curating, and evaluating graphs that can serve as a starting point for new multi-omics studies.

## Graphical Abstract

**Graphical Abstract:**
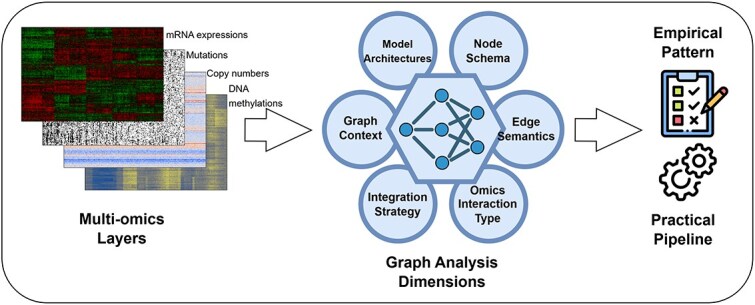

## Introduction

Modern high-throughput technologies now allow us to profile biological systems at multiple molecular layers, including the genomes, epigenomes, transcriptomes, proteomes, and metabolomes, often within the same cohort [1–4]. These multi-omics datasets promise a more complete view of how regulatory, signaling, and metabolic processes interact to shape cellular states and disease phenotypes. However, they also pose substantial analytical challenges. Different omics layers exhibit distinct noise properties and dynamic ranges: mRNA-sequencing (mRNA-seq) data are count-based and affected by sequencing depth, overdispersion, and dropout; chromatin accessibility profiles are sparse and often close to binary at individual loci; proteomic measurements may contain antibody background, peptide detection bias, or missing values; methylation signals are often reported as beta values between 0 and 1, creating a bounded scale with different variance properties; and metabolomic features can span several orders of magnitude in concentration. As a result, variation across modalities may reflect technical scale, detection sensitivity, or measurement chemistry rather than shared biological structure. In addition, feature spaces are large and heterogeneous, and samples are often only partially profiled or drawn from small cohorts [5, 6]. Simply concatenating features across modalities rarely preserves biological structure and can degrade downstream inference by prioritizing high-variance technical effects over shared biology.

A wide range of multi-omics integration strategies have therefore been proposed, spanning classical statistical methods, matrix factorization, kernel and graph-based approaches, and more recent deep learning models [7]. These methods differ in when and how they combine information across layers, what they treat as the basic unit of analysis, and how strongly they rely on prior biological knowledge versus purely data-driven patterns. Despite this diversity, a common theme is that molecular biology is inherently relational [8]: genes co-regulate one another, proteins interact in complexes and pathways, chromatin regions co-access or loop in three dimensions, and cells communicate within tissues. Capturing these relationships explicitly is often crucial for robust integration and meaningful interpretation.

Graphs provide a natural language for representing such relational structure [9–11]. In a graph, nodes can correspond to genes, proteins, metabolites, cells or samples, and edges can encode physical interactions, regulatory links, pathway membership, co-expression, similarity, or spatial proximity. Graph-based deep learning, particularly graph neural networks (GNNs) and related architectures, can then operate directly on these graphs to propagate information along edges, aggregate signals from neighbors, and learn representations that respect known or inferred structure. These frameworks are flexible enough to support both within-omics and cross-omics connectivity, to mix knowledge-driven and data-driven edges, and to adapt to bulk, single-cell, and spatial multi-omics settings.

In recent years, many graph-based deep learning methods have been proposed for multi-omics integration, but they vary widely in how they build graphs and how those graphs are used within the model. Some methods focus on homogeneous graphs, where all nodes share the same type and relations are modeled within a single domain, while others employ heterogeneous graphs that explicitly encode multiple node and edge types to capture richer cross-level biological interactions. Edge definitions range from curated interaction networks to learned similarity measures, and integration strategies span tightly coupled joint graphs to modular architectures that link modality-specific graphs at later stages. These design choices have important consequences for interpretability, data requirements, scalability, robustness to noise, and missing modalities, and ultimately for the kinds of biological questions that can be addressed.

In this review, we adopt a graph-centric perspective on multi-omics deep learning. Rather than primarily cataloging model architectures, we emphasize how graphs are defined, constructed, and curated, and how these design choices influence downstream performance. We organize existing methods along several key axes, including graph granularity, edge semantics, interaction type, integration strategy, graph context, and model architecture, across bulk, single-cell, and spatial settings. By comparing approaches within this unified framework, we aim to clarify common patterns, highlight trade-offs, and identify underexplored design spaces.

Finally, drawing on these comparisons, we propose a practical pipeline for building graphs for multi-omics integration, from defining the scientific objective and node view, through edge construction and quality control, to model training and evaluation. Our goal is to provide both a structured overview of the field and concrete guidance for practitioners who wish to design or adapt graph-based deep learning methods for their own multi-omics data.

## Foundations of multi-omics profiling

### Different types of omics

The term omics includes a spectrum of large-scale biological disciplines that collectively characterize and quantify the molecules driving cellular function. Each omics layer provides a complementary snapshot of biological organization, ranging from DNA sequences to metabolic products, and together they build a more complete and systems-level picture of how living systems function.

#### Genomics

Genomics is the study of the complete DNA content of an organism, including gene sequences, non-coding regions, structural variants, and copy-number changes. It provides the foundational blackprint of genetic information on which other omics layers depend (e.g. which genes are present and what regulatory sequences can control their expression). Key genomic technologies include genotyping arrays [12], exome sequencing [13], and whole-genome sequencing, now often performed with third-generation long-read platforms [1]. These tools enable large-scale studies such as GWAS [14] to link genetic variation with traits, although many associated variants fall in non-coding regions and their functional roles remain unclear [15].

#### Transcriptomics

Transcriptomics is the study of the complete set of RNA transcripts, the transcriptome, produced by the genome in a cell, tissue, or organism under specific conditions. This includes messenger RNA (mRNA), as well as diverse non-coding RNAs such as long non-coding RNA (lncRNA), and microRNA (miRNA). The primary technology in this field is RNA sequencing (RNA-seq), which enables both qualitative analyses (e.g. transcript and isoform identification) and quantitative measurements of transcript abundance, even from small or low-input samples [2]. By profiling changes in RNA levels, transcriptomics reveals gene expression dynamics and provides a snapshot of the active biological program at the RNA level [16].

#### Epigenomics

Epigenomics refers to the genome-wide study of epigenetic modifications, including DNA methylation, histone modifications, chromatin accessibility, and other structural features that regulate gene expression without changing the underlying DNA sequence [17]. In this sense, the epigenome guides how the genome is used, and shifts in epigenomic states play key roles in development, differentiation, and disease. Major technologies include bisulfite sequencing for DNA methylation profiling, chromatin immunoprecipitation sequencing (ChIP-seq) for mapping histone modifications [18], and assay for transposase-accessible chromatin using sequencing (ATAC-seq) for measuring chromatin accessibility [3], which together enable comprehensive analysis of the epigenetic landscape [19, 20].

#### Proteomics

Proteomics investigates the proteome, the complete set of proteins expressed in a cell, tissue, or organism at a given time, including their post-translational modifications, interactions, and functions. Because proteins act as the main effectors of cellular activity, such as enzymes, structural components, and signaling molecules, proteomics provides a functional readout of genomic and transcriptomic programs. Modern proteomics mainly relies on mass spectrometry–based approaches, including both label-free and isotope-labeled quantification, as well as affinity-based technologies such as protein microarrays and immunoprecipitation assays, to identify, quantify, and characterize proteins and their modifications [21–23].

#### Metabolomics

Metabolomics is the study of small-molecule metabolites, such as substrates, intermediates, and products of biochemical reactions, within a biological system, collectively referred to as the metabolome. Because metabolites integrate the effects of gene expression, enzyme activity, and environmental exposures, metabolomics often provides a close, real-time readout of cellular physiology. Common analytical platforms include mass spectrometry (MS)-based methods such as gas chromatography–mass spectrometry (GC-MS), liquid chromatography–mass spectrometry (LC-MS), and capillary electrophoresis–mass Spectrometry (CE-MS), as well as nuclear magnetic resonance spectroscopy, which enable sensitive detection and quantification of diverse metabolites in biological samples [24, 25].

#### Metagenomics

Metagenomics refers to the culture-independent sequencing of genetic material from microbial communities, enabling the profiling of microbial composition, diversity, and functional potential [26]. In multi-omics studies, it provides a microbial layer that can be integrated with host transcriptomics, metabolomics, metatranscriptomics, proteomics, and immune profiling to study host–microbiome interactions. Recent advances include deeper shotgun metagenomics, strain-resolved analysis, metagenome-assembled genomes, and long-read sequencing platforms such as Nanopore and PacBio, which can improve genome assembly and microbial resolution [27]. Dual-sequencing strategies further extend this direction by jointly profiling host and microbial activity from the same biological context; for example, dual RNA-seq enables simultaneous measurement of host and pathogen transcriptomes [28, 29], while recent spatial metatranscriptomic approaches capture host gene expression together with microbial signals in intact tissue sections [30].

#### Other omics

Beyond these core domains, numerous specialized omics branches further expand the molecular landscape, including lipidomics [31], which focuses on lipid metabolism; glycomics [32], which examines carbohydrate structures; and interactomics [33], which maps molecular interaction networks. These complementary datasets are increasingly integrated into multi-omics frameworks to uncover the complex interplay among genetic, regulatory, and environmental factors in health and disease.

### Measurement scales and resolutions: bulk, single-cell, and spatial approaches

Molecular information can be measured at different levels of granularity and in different biological contexts. The measurement scale defines which biological unit is being profiled (e.g. a tissue, a group of cells, a single cell, or a subcellular region), while the resolution reflects how precisely the method can distinguish individual components or spatial locations. Bulk omics operate at a population-level scale, averaging signals across many cells and therefore providing low resolution. Single-cell omics increase the resolution to the level of individual cells, enabling the study of cell-to-cell variation. Spatial omics extend this further by adding spatial resolution, mapping molecular profiles to their physical locations within tissue architecture. Together, these scales and resolutions determine the depth and contextual detail that multi-omics analyses can reveal.

#### Bulk multi-omics

Bulk multi-omics refers to the integrative analysis of multiple molecular layers measured from a pooled population of cells within a biological sample. Unlike single-cell multi-omics, which resolves molecular information at the level of individual cells, bulk multi-omics captures the average molecular signal across all cells in a tissue, blood sample, or cultured population. This approach is widely used in systems biology and clinical research to obtain a comprehensive, multi-layered view of biological processes, disease mechanisms, and patient heterogeneity at the tissue or organismal level.

The generation of bulk multi-omics data involves extracting biomolecules such as DNA, RNA, proteins, and metabolites from homogenized tissues or pooled samples, followed by modality-specific assays. For each modality, these assays produce a high-dimensional sample-by-feature matrix measured on the same or an overlapping set of samples, which typically requires normalization and joint analysis across modalities to reconcile differences in scale, distribution, and missingness. Although bulk data lack single-cell resolution and can obscure signals specific to particular cell types, it offers a scalable and cost-effective strategy for large cohorts. Public datasets like TCGA [34] and METABRIC [35] exemplify this approach and support applications in disease subtyping, biomarker discovery, and systems-level analysis using statistical, machine learning, or graph-based methods.

Bulk transcriptome profiling (e.g. RNA-seq) has been widely adopted, making transcriptomics by far the most commonly used layer in bulk multi-omics integration. Epigenomic measurements, such as DNA methylation and chromatin accessibility, along with genomic variation profiles [e.g. single-nucleotide variants and copy-number variants (CNVs)], are also routinely included, with pairwise combinations of transcriptomics and epigenomics or genomics appearing particularly often. Many studies further extend these designs by incorporating additional modalities, such as proteomics, metabolomics, or metagenomics, so that three or more omics layers are frequently analyzed together to provide a more comprehensive view of the regulatory and genetic mechanisms underlying disease [36].

#### Single-cell multi-omics

Single-cell omics is a field of biology that uses advanced technologies to analyze and understand complex biological systems at the individual cell level. It involves technologies that profile molecular layers such as DNA, RNA, proteins, and epigenetic states in individual cells to reveal insights into cell function, development, heterogeneity, and disease. Single-cell multi-omics extends this by profiling multiple molecular layers at single-cell resolution, either jointly within the same cell or by integrating closely matched but separately measured single-cell modalities. Instead of averaging signals across a tissue, single-cell multi-omics looks at each individual cell to see all its unique characteristics and understand how those different cells work together in a tissue. This fine resolution has transformed our understanding of cell development, cancer progression, immune responses, and tissue organization.

At its core, the single-cell multi-omics workflow begins with isolating individual cells or nuclei through technologies like droplet microfluidics [37], fluorescence-activated cell sorting (FACS) [38], or laser capture microdissection [39]. Once isolated, distinct or combinatorial assays are used to measure various molecular layers. For instance, scRNA-seq [40] quantifies mRNA transcripts to map gene activity, while scATAC-seq [41] identifies open chromatin regions that mark regulatory elements. DNA methylation patterns can be profiled at single-cell resolution using scBS-seq [42], and surface proteins can be measured alongside RNA using antibody-tagged methods like CITE-seq [43] or REAP-seq [44]. More recently, multi-modal techniques such as SHARE-seq [45], SNARE-seq [46], and 10x Multiome [47] allow simultaneous measurement of transcriptome and chromatin accessibility from the same cell, bridging the gap between gene regulation and expression.

Unlike bulk datasets that produce stable and dense signals, single-cell data are sparse, noisy, and high-dimensional [48, 49]. Each omics layer presents unique biases and dropout rates, requiring sophisticated computational pipelines for normalization, batch correction, and integration. Moreover, many experimental designs generate unmatched multi-omics, where different modalities are measured in separate but similar cell populations, so correspondences between cells across modalities must be inferred computationally. Methods drawing on matrix factorization, manifold alignment, optimal transport, and deep neural networks have been developed to reconcile modality-specific feature spaces and align cellular states across assays [50, 51].

The power of single-cell multi-omics lies in its ability to reconstruct cellular hierarchies, map differentiation trajectories, and identify cell-type-specific regulatory networks that govern complex phenotypes. Initiatives like the Human Cell Atlas [52] aim to use such technologies to build a complete molecular reference of all human cell types.

#### Spatial multi-omics

Spatial multi-omics couples molecular profiling with tissue coordinates, measuring multiple omics layers while preserving the intact spatial organization of the tissue. Unlike traditional sequencing methods that dissociate cells and lose spatial organization, spatial multi-omics preserves the positional information of molecules, linking molecular signatures to their precise locations in tissues. This spatially resolved view reveals how tissue architecture and microenvironmental organization constrain and guide cellular arrangement and interactions, shaping local cell–cell communication and ultimately influencing development and disease progression at both cellular and subcellular scales.

Spatial multi-omics technologies have rapidly progressed from transcriptome-only approaches to multi-layered platforms that capture multiple molecular modalities. Spatial transcriptomics methods, including 10x Genomics Visium [4], Slide-seq [53], and MERFISH [54], measure RNA abundance across tissue sections. In parallel, spatial epigenomics techniques such as spatial ATAC-seq [55] and spatial CUT&Tag [56] profile chromatin accessibility and histone modifications in situ. The integration of proteomics, enabled by imaging mass cytometry and multiplexed immunofluorescence, further expands this framework to capture spatially resolved protein expression. More recent platforms, including DBiT-seq [57] and seqFISH+ [58], enable the simultaneous measurement of RNA, protein, and epigenetic states within the same tissue section, providing a unified view of cell–cell interactions and local microenvironments.

Computationally, spatial multi-omics datasets are represented as matrices that encode molecular measurements along with spatial coordinates, often at spot- or cell-level resolution. In many platforms, spatial measurements are taken on fixed capture regions (spots) rather than individual cells. Since each spot covers a small tissue neighborhood, the resulting signal is typically a composite of several cells’ contributions. Integration across modalities requires mapping measurements to a common spatial frame by aligning coordinates across sections or assays and reconciling differences in spatial resolution. It also requires normalization of signal intensities across assays and explicit modeling of spatial dependencies between neighboring cells or spots. Together, these steps support integrated analysis that links molecular variation to tissue organization and local cell interactions.

Spatial multi-omics is transforming the study of biology and disease by linking molecular readouts to physical locations. Capturing region-specific gene expression together with chromatin or protein states reveals how local microenvironments shape gene regulation and cellular interactions. Such spatial insight has uncovered new dimensions of tissue development, tumor evolution, immune responses, and cancer progression, including patterns of invasion and therapeutic resistance [59–61]. Large-scale initiatives such as Human BioMolecular Atlas Program (HuBMAP) [62] and Human Tumor Atlas Network (HTAN) [63] now employ spatial multi-omics to map human organs and diseases at unprecedented resolution, paving the way for comprehensive molecular atlases that integrate structure and function.

## Analysis dimensions

**Figure 1:**
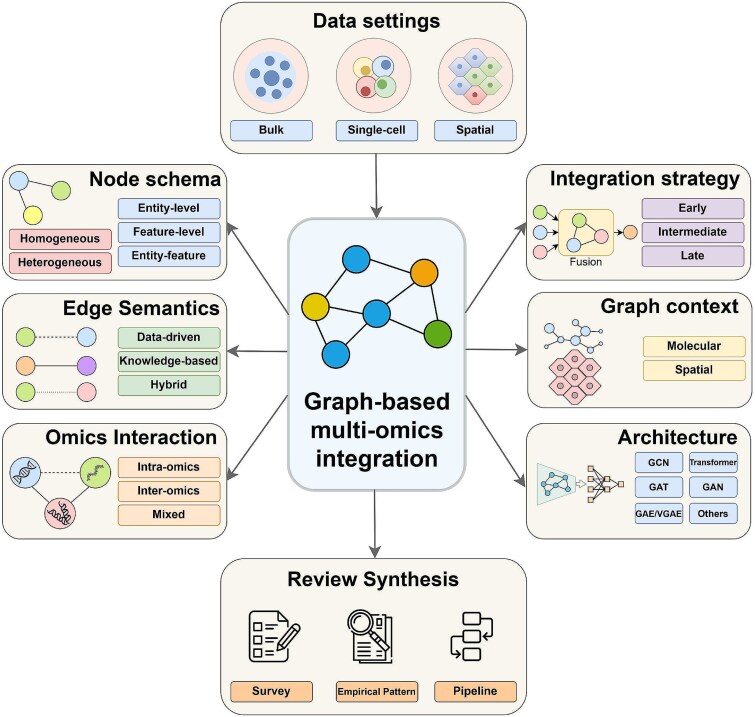
Conceptual framework of the review. The review organizes graph-based multi-omics integration methods across bulk, single-cell, and spatial settings by graph design choices, deep learning architectures, empirical design patterns, and practical pipeline considerations.

Graph-based modeling has emerged as a powerful paradigm for multi-omics integration, allowing complex biological systems to be represented as interconnected networks rather than independent data tables. In this view, nodes can represent biological units such as genes, proteins, metabolites, cells, or patients, while edges encode known or inferred relationships, allowing learning algorithms to leverage biological priors such as protein–protein interactions (PPIs), regulatory links, and pathway structure. By applying geometric deep learning (e.g. GNNs), it becomes possible to learn informative node and graph-level embeddings that naturally integrate heterogeneous omics measurements while respecting the non-Euclidean structure of biological networks. These models propagate information through the graph to uncover multi-layered dependencies across modalities, improve prediction and clustering performance, and provide biologically interpretable insights into complex traits and diseases. In the following subsections, we analyze the graphs used in deep learning-based multi-omics integration from different perspectives. These perspectives and their corresponding categories are illustrated in Fig. 1. Tables 2–4 present the surveyed graph-based integration models in bulk, single-cell, and spatial settings, respectively, grouped according to the perspectives discussed in this section.

### Node schema

Nodes in multi-omics graphs can be described along two complementary axes. The first is node granularity, i.e. what a node represents. In bulk, single-cell, and spatial settings, the primary measurement units are samples, cells, and cells/spots, respectively; we refer to these units as “entities.” The molecular measurements attached to each entity form the “features” of each omics layer. From this perspective, graphs can be categorized as entity-level, feature-level, or entity–feature. Entity-level graphs place entities as nodes and connect them by profile similarity, whereas feature-level graphs place molecular features as nodes and connect them by functional or statistical relationships. Entity–feature graphs jointly include both entities and features as nodes, with edges encoding observed measurements between them.

A second axis is node-type composition, i.e. how many distinct node types a graph contains. Homogeneous graphs include a single node type, such as a cell–cell graph or a gene–gene graph. Heterogeneous graphs include multiple node types within the same structure (e.g. entities together with features, multiple entity types, or multiple feature types), and connect them with edges that can span types. This distinction is useful because homogeneous graphs primarily model relationships within a single view, whereas heterogeneous graphs can explicitly encode cross-type couplings; in practice, many multi-omics frameworks use one or both designs depending on the task. We next provide a more detailed discussion of each classification.

#### Node granularity

Node granularity is often the first practical design decision because it determines what the model embeds: either entities such as samples, cells, or spots, or molecular features such as genes, proteins, and peaks. This choice then controls where supervision and evaluation naturally apply, since labels are typically defined at the entity level, whereas mechanistic interpretations and link-level hypotheses are usually defined at the feature level. It also shapes how edges are constructed and how outputs should be interpreted, ranging from similarity neighborhoods among entities to functional or regulatory couplings among features.

### Entity-level graphs

**Figure 2:**
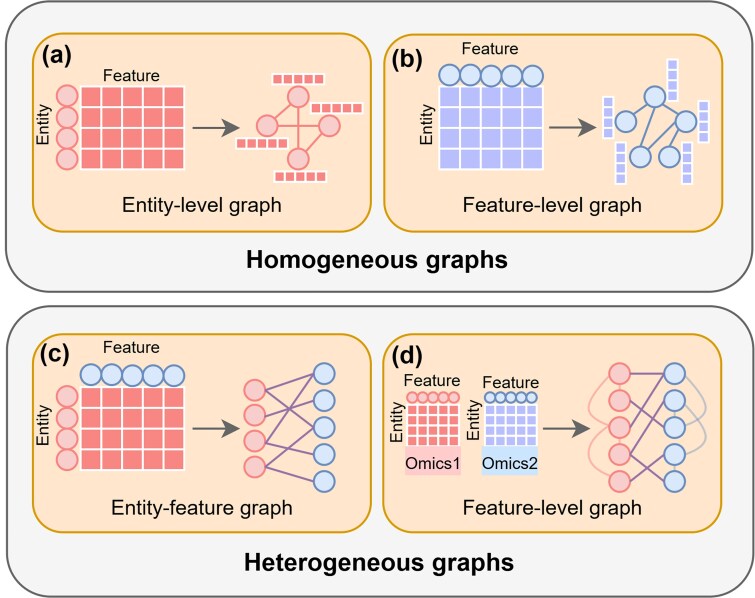
Node schema. (a) Entity-level graph with nodes defined as entities. (b) Feature-level graph with nodes defined as features. (c) Entity-feature bipartite graph linking entities to features through measured values. (d) Cross-omics feature-level heterogeneous graph linking features across multiple omics modalities. Panels (a-b) show homogeneous graphs, while panels (c, d) show heterogeneous graphs.

Entity-level graphs, where nodes represent biological units such as samples, cells, or spatial spots and edges encode relationships between them, are widely used in multi-omics integration because they capture population-level patterns, enabling comparison and grouping based on overall molecular profiles (Fig. 2a). These graphs are particularly effective for tasks such as classification, clustering, and trajectory inference, where capturing similarities among entities is essential. They are also relatively easy to construct using well-established similarity metrics (e.g. Euclidean distance, cosine similarity, Pearson correlation), which can be applied directly to multi-omics feature vectors. Because each node corresponds to a single biological unit, multiple omics layers can be incorporated as complementary views or channels of the same node. Such integration is well-suited for early or intermediate fusion strategies and scales efficiently to downstream tasks like supervised learning, where entity-level embeddings can be directly linked to labels (e.g. disease subtypes). As a result, entity-level graphs are commonly employed in multi-omics integration frameworks. This includes patient–patient networks in bulk multi-omics approaches (e.g. MOGONET [64], MoGCN [69]), cell–cell networks in single-cell integration methods (e.g. GCN-SC [94], scGCN [91]), as well as spatial graphs in spatial omics frameworks (e.g. SpatialGLUE [111], SSGATE [112]).

However, entity-level graphs also present several challenges and limitations. The curse of dimensionality is a major issue, especially in bulk or clinical omics, where the number of samples is much smaller than the number of features [127]. Additionally, different omics types often have heterogeneous scales, distributions, and missing modalities, making it difficult to compute a unified similarity metric across entities [128]. The choice and normalization of the similarity metric critically influence graph structure and downstream results. In single-cell and spatial omics, high noise and sparsity, such as dropout effects in scRNA-seq or missing spatial features, can distort similarity calculations [129]. More fundamentally, entity-level graphs lack biological interpretability at the molecular level, as they do not model direct functional relationships among features. As such, they are not well suited to capturing gene regulatory interactions, PPIs, or pathway-level mechanisms. This profile-centric integration focuses on overall entity similarity rather than capturing the inter-feature dependencies that are often key to understanding the underlying biology.

### Feature-level graphs

Feature-level graphs are particularly suited to uncovering biological mechanisms and regulatory programs within and across omics layers. Here, nodes correspond to molecular features such as genes, transcripts/isoforms, proteins, metabolites, CpG sites, chromatin peaks, enhancers, or transcription factors, and edges capture functional coupling, regulation, or statistical association between features (Fig. 2b). Importantly, these edges may be intra-omics (e.g. gene–gene co-expression, protein–protein interactions) or inter-omics (e.g. peak–gene, TF–target, methylation–gene, miRNA–mRNA), enabling models to learn coordinated programs that span modalities. This shifts the analytical focus from comparing whole entities to identifying interacting feature modules, supporting tasks such as network inference, functional annotation, module discovery, and mechanism-driven integration.

Feature-level graphs also naturally support inter-omics integration when constructed across different omics types. For example, bipartite graphs can be used to connect features from transcriptomics and epigenomics (e.g. genes and regulatory elements), allowing the model to learn cross-modal regulatory relationships. Similarly, existing biological knowledge, such as transcription factor binding networks, PPI databases, or curated pathways, can be incorporated into the graph structure, making feature-level graphs a natural choice for knowledge guided integration frameworks. Feature-level networks are observed in many multi-omics integration frameworks, as demonstrated in methods, such as PTNET [65], EMOGI [66], and GLUE [93].

However, feature-level graphs come with their own set of challenges. Constructing such graphs typically requires large sample sizes to robustly estimate inter-feature relationships, especially in single-cell and spatial omics where data sparsity is prevalent. The quality of the feature graph is highly dependent on the choice of similarity metric, normalization strategy, and prior knowledge sources. Additionally, these graphs do not directly align with labels or phenotypes at the entity level, which can limit their effectiveness in supervised prediction tasks unless followed by an additional aggregation or embedding step.

### Entity–feature graphs

Entity–feature graphs represent entities and omics features as separate node sets connected by measurement edges, effectively turning the data matrix into a bipartite or multipartite graph (Fig. 2c). This construction supports joint learning of entity embeddings for downstream prediction and feature embeddings for mechanistic interpretation, since message passing propagates information between entity states and molecular programs. In multi-omics settings, multiple omics layers can attach to the same entity layer, allowing integration through entity–feature connections instead of collapsing everything into one similarity metric; if a modality is absent, its edges are simply not added. Because the graph explicitly encodes that features are active in which entities, it is naturally suited for tasks that benefit from both population structure and feature attribution, such as stratification with marker discovery or spatial domain identification with localized gene programs.

In practice, constructing entity–feature graphs is rarely seen in bulk multi-omics because entities are individuals, and relationships between individuals and omics features do not have a clean, reusable interpretation beyond the observed profiles. In single-cell and spatial omics, where entities are cells or spots, entity–feature links are easier to define and often more meaningful, since they can directly reflect measured expression, accessibility, or protein abundance. Many methods including scMoGNN [92], scMoFormer [95], and DeepMAPS [97] build cell–feature graphs linking cells to genes, peaks, and proteins, where edge weights are defined by the corresponding observed expression, accessibility, or protein measurements.

However, these graphs can be extremely large and sparse, with computational cost scaling with the number of nonzero measurements, and performance can be sensitive to preprocessing choices that directly determine edge weights and connectivity. Since entity–feature edges reflect observed data rather than functional priors, the base graph may capture co-occurrence structure more than direct molecular interactions, and often benefits from augmentation with feature–feature biological edges. Moreover, technical noise, library size effects, and modality imbalance can dominate message passing unless normalization and modality-aware modeling are carefully designed.

#### Node-type composition

Node-type composition specifies whether a graph contains a single node type or multiple node types within the same structure. This design choice determines which interactions can be represented explicitly, ranging from within-type similarity neighborhoods to cross-type biological couplings. We distinguish between homogeneous and heterogeneous graphs and summarize when each is preferred.

### Homogeneous graphs

Homogeneous graphs contain a single node type, so all nodes represent the same kind of object (Fig. 2a and b). In multi-omics integration, common examples include entity-only graphs such as sample–sample networks in bulk studies, cell–cell graphs in single-cell analysis, and spot–spot graphs in spatial assays, where edges reflect similarity derived from multi-omics profiles. Homogeneous designs also appear as feature-only graphs, such as gene–gene networks built from co-expression, pathways, or protein–protein interactions, or peak–peak graphs built from co-accessibility, where nodes are molecular features and edges encode functional or statistical coupling. Many bulk, single-cell, and spatial frameworks adopt homogeneous graphs at either the entity or feature-level. For instance, MOGONET [64], scGCN [91], and SpatialGLUE [111] construct homogeneous entity-level graphs, whereas GOAT [74], TEMINET [81], and scMID [104] operate on homogeneous feature-level graphs.

Homogeneous graphs are often simpler to define, store, and scale, and they fit standard message passing architectures without requiring type-specific parameters. They also yield embeddings that are easy to interpret because every node lives in the same semantic space. However, they cannot explicitly represent cross-type structure, so interactions like peak–gene regulation or gene–protein mapping must be injected indirectly through feature fusion, shared latent spaces, or multi-relational edge design over a single node type. As a result, some mechanistic relationships may be diluted or lost when heterogeneous biology is compressed into one node space.

### Heterogeneous graphs

Heterogeneous graphs include more than one node type, which makes them well-suited to multi-omics settings where different biological objects must be represented jointly (Fig. 2(c,d)). Nodes may correspond to entities such as cells, samples, or spatial spots, and/or to molecular features such as genes, chromatin regions, proteins, and methylation sites. Unlike homogeneous graphs that connect a single node type through similarity or neighborhood structure, heterogeneous graphs represent cross-type relations explicitly. A typical example is connecting a cell node to gene nodes using the observed expression profile, with edge weights proportional to expression levels. This provides the model with richer propagation pathways, including chains that traverse modalities rather than remaining within one node space.

Entity–feature graphs are therefore heterogeneous by construction, since edges link entities to omics features using the observed measurement matrix or learned/defined incidence weights. Methods such as scMoGNN [92], MarsGT [99], and SuperGLUE [108] build heterogeneous networks that combine entity nodes, typically cells, with feature nodes such as genes, peaks, and proteins to couple modalities via typed edges instead of relying only on a homogeneous cell–cell graph. Heterogeneous graphs can also arise within a single granularity. Some spatial frameworks may build an entity-level spot–cell graph that links each spot to similar cells based on their molecular profiles. At the feature level, many bulk, single-cell, and spatial methods construct multi-type feature graphs in which distinct feature spaces (i.e. different omics types) are linked by prior knowledge or statistical associations, for example coupling genes with peaks or methylation loci. PTNET [65], GLUE [93], and MultiGATE [116] are examples that use heterogeneous feature graphs to connect features across omics layers.

Heterogeneous graph construction starts with selecting which biological objects will appear as node types, and then choosing how to encode their relationships, which can be as simple as a single cross-type edge set or extended to multiple typed relations with distinct weighting and normalization. Relations may be imported from multi-omics priors, such as peak–gene regulation, gene–protein correspondences, or ligand–receptor interactions, or learned from data through latent alignment, mutual nearest neighbors, or attention-derived edges. The advantage is expressivity: the model can keep object identities separate and support type-aware interpretation, for example prioritizing peaks linked to a transcriptional program or tracing cross-modal paths in spatial settings. The trade-off is a larger and more sensitive model, with more relation-dependent design choices and a higher risk from noisy or dense edges, which calls for sparsification and strong regularization.

### Edge semantics

In graph-based representations of omics data, edge semantics refer to the nature and interpretation of the edges: the connections between nodes. While nodes encode biological units such as samples, cells, spots, or molecular features, the edges define how these units are related. The edge type is therefore pivotal in shaping the kind of information the graph encodes and influences how this information propagates across nodes and affects the final representation.

Depending on the biological question and the assay types being integrated, the graph may use different edge semantics. This subsection categorizes and examines the common edge semantics used in omics graphs, highlighting their construction methods, assumptions, and implications for integration and interpretation.

#### Data-driven graphs

**Figure 3:**
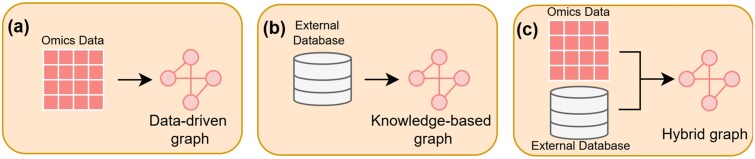
Edge semantics. (a) Data-driven graph constructed solely from omics data. (b) Knowledge-based graph constructed from external knowledge source(s). (c) Hybrid graph constructed from input data and external source(s).

Data-driven graphs are constructed solely from the input data, without relying on any external sources (Fig. 3a). These graphs can capture various relationships, such as similarity-based connections (e.g. correlations or distances in expression space), spatial proximity (e.g. physical adjacency of tissue spots), or dependencies that are inferred from the data by the model (e.g. through attention mechanisms or graph learning techniques).

Similarity-based graphs are a common form of data-driven graphs constructed by quantifying pairwise relationships between biological entities, based on their omics profiles. These relationships are typically measured using similarity or distance metrics like cosine similarity, Pearson or Spearman correlation, or Euclidean distance. Advanced affinity functions such as Gaussian kernels or locally adaptive kernels can capture more complex relationships. Graph topology is often established using strategies like k-nearest neighbors (k-NN), $\epsilon $-radius, or mutual nearest neighbors (MNN), enabling flexible yet efficient construction. MOGONET [64], MOGLAM [72], and Subtype-MGTP [79] employ cosine similarity, whereas SUPREME [71] and MOGAT [76] use Pearson correlation coefficient. MoGCN [69], hMKL [130], DeepMoIC [77], and MOSEGCN [80] apply Euclidean distances combined with Gaussian kernels to define edges. While conceptually simple and widely used in multi-omics tasks like patient stratification or clustering, such graphs are often limited to static, local, and noise-sensitive representations.

Beyond similarity-based graphs, data-driven approaches can learn graph structure directly from the data, allowing them to capture nonlinear and higher-order dependencies. For example, MOGLAM [72] constructs an adaptive graph for each modality by learning edge weights from cosine-similarity-based priors, guided by a smoothness-promoting Dirichlet energy loss. Such learned-graph frameworks may reveal latent biological structures that static similarity metrics often miss.

In the context of spatial multi-omics, spatial graphs form a central class of data-driven graphs. In spatial graphs, nodes usually correspond to spatial spots or individual cells, and edges encode neighborhood relations based on their coordinates or on the underlying array layout. Spatial integrative models use these graphs to inject spatial dependence and tissue organization into the learning process, encouraging representations that reflect how nearby locations influence each other. Common constructions rely on Euclidean distances, optionally combined with Gaussian or other kernel functions and then connect spots or cells through schemes such as k-NN or MNN [111, 112, 131]. Importantly, many models also construct complementary graphs from omics-derived feature relationships or histology features, and either pair these with the spatial graph or keep them as separate graphs that are combined at the embedding level rather than fused into a single topology [114, 118, 119, 132].

Data-driven graph construction offers several advantages: it is adaptable to diverse datasets, does not require external annotations, and can uncover emergent structures from raw measurements. However, these graphs also have limitations. Because data-driven graphs are constructed from numerical data, their quality depends strongly on the input data, the similarity or association metric used to compare profiles, and the criteria used for edge selection. Errors at any of these stages can mislead downstream learning and interpretation. In addition, data-driven graphs may lack biological interpretability, as they rely on statistical associations rather than mechanistic insight. Their sensitivity to noise, hyperparameters (e.g. $k$ in k-NN), and the selected similarity measure or learning algorithm can introduce spurious or biased edges. Furthermore, when graphs are static rather than learned or updated during training, they may fail to capture evolving relationships or global dependencies in high-dimensional and heterogeneous omics data.

#### Knowledge-based graphs

Knowledge-based graphs are constructed using curated biological knowledge rather than data-driven similarities (Fig. 3b). They encode known regulatory, functional, or biochemical relationships derived from experimental studies, literature, and expert annotations. Such information is gathered from well-established biological databases that integrate diverse experimental and computational evidence, combining high-throughput omics data with manual curation and validation. These knowledge-based graphs enable biologically meaningful alignment across omics layers, capturing interactions that reflect established mechanisms rather than purely statistical associations.

External information from curated databases can be converted into graphs by treating molecular features such as genes, proteins, and metabolites as nodes, and adding edges for experimentally supported or annotated relationships. These edges can represent tissue or compartment context, subcellular localization, pathway and gene ontology relationships, protein complex membership, known ligand–receptor pairs, and enhancer–promoter links, depending on the biological context. Edge attributes such as direction, confidence score, and interaction type can further encode whether a connection reflects regulatory influence, functional association, or biochemical coupling within the graph. Common examples of external biological knowledge include PPI networks, which capture physical and functional associations among proteins. Models such as EMOGI [66] utilize these networks to build comprehensive linkage maps by integrating PPI data from multiple curated sources, including CPDB [133], STRING-db [134], PCNet [135], IRefIndex [136], and MultiNet [137]. Post-transcriptional regulatory networks model directed interactions from miRNAs to their target mRNAs. For example, PTNet [65] constructs a bipartite graph of miRNA to mRNA pairs obtained from TargetScanHuman [138]. Other frameworks, such as GLUE [93], leverage genomic proximity and epigenomic annotations to link chromatin-accessible regions or methylated loci to nearby genes using promoter overlaps, distance-based weighting, or experimentally supported interactions (e.g. pcHi-C [139], eQTL [140]). Together, these strategies capture detailed representations of molecular and regulatory architectures across biological layers.

By embedding mechanistic insights into the graph structure, knowledge-based graphs allow models to propagate information along biologically meaningful edges, enhancing interpretability and constraining learning to validated pathways. However, they may be limited by incomplete or biased curation, often favoring well-studied entities and lacking context-specific or dynamic regulation [141, 142]. Many resources also provide static interaction maps, which may not generalize across cell types or conditions.

#### Hybrid graphs

Omics interaction networks can also be constructed in a hybrid manner, combining data-driven structure with knowledge-based priors, such as curated molecular interactions, pathway and ontology memberships, regulatory programs and motifs, cell-type markers, and known complexes or pathways (Fig. 3c). These networks can further incorporate biological constraints and hierarchies, directionality or causal signs (e.g. activation or repression), temporal or developmental ordering, and phenotype or clinical covariates, which can be encoded either as additional node features or as global sample-level features shared across the graph. Many databases also provide confidence scores or levels of supporting evidence for each interaction, which can be used to weight edges or reflect uncertainty. Overall, this produces graphs that follow established biology while still adapting to signals in the data, improving interpretability, robustness and generalization.

Hybrid graphs can be constructed in multiple, complementary ways. One approach may start with a data-driven network, for example based on similarities, co-occurrence, or statistical associations, and then augment or refine it using curated biological knowledge. Another co-encoding strategy may feed raw data and priors together (pathways, PPIs, gene sets) into a joint encoder so the graph emerges directly from learned representations. Alternatively, one can use parallel layers for data and prior knowledge and link them with learnable coupling, or initialize from a knowledge graph and adapt its edges using the data. There is no single recipe; the choice depends vastly on the upstream/downstream task and the designer’s goals. For example, MODIG [68] makes a hybrid graph by taking a gene network from databases (PPI [134], KEGG [143], Gene Ontology (GO) [144]) and adding extra gene–gene edges learned from data (co-expression and sequence similarity), keeping each as its own layer. scMoGNN [92] builds a hybrid cell–feature bipartite graph with data-driven weighted edges from the observed modality matrix and, for gene-based tasks, augments the feature side with knowledge-based gene–gene links from MSigDB [145] hallmark gene sets to inject biological priors.

Hybrid graphs offer strong biological plausibility and interpretability by anchoring edges in curated priors while still capturing cohort-specific structure and novel associations from data-driven similarities. They can regularize learning in low-sample or noisy settings, improve generalization across cohorts, and mitigate sparsity by filling gaps where one source is weak. However, they introduce fusion complexity, such as how to weight, threshold, or reconcile layers, risk bias or staleness from outdated or uneven knowledge bases, and may overconstrain models, which can dampen the discovery of new biology if priors dominate. Conflicting edges and heterogeneous confidence scores require calibration, performance can be sensitive to hyperparameters (e.g. layer weights), and the multi-layer construction can increase computational and maintenance costs, including versioning of priors and periodic updates.

### Omics interaction type

In multi-omics integration, the type of omics interaction determines whether graph edges represent relationships within a single modality (intra-omics), across different modalities (inter-omics), or a combination of both (mixed). This classification guides graph construction and influences how biological information is fused across molecular scales. This subsection describes each interaction type in detail, emphasizing their biological relevance, modeling strategies, and practical implications in bulk, single-cell, and spatial omics settings.

#### Intra-omics graphs

**Figure 4:**
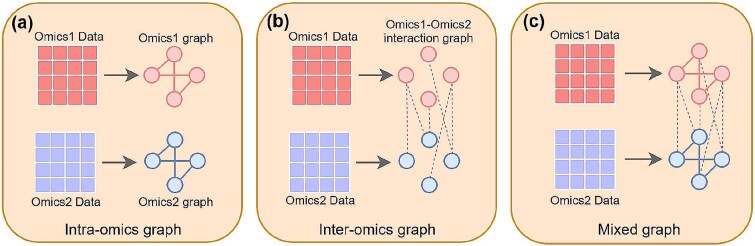
Omics interaction graphs. (a) Two independent intra-omics graphs derived from two omics layers. (b) Inter-omics graph defined only by cross-layer connections. (c) Mixed graph combining intra-omics graphs with additional inter-omics interaction edges.

Intra-omics graphs represent relationships within a single omics layer (Fig. 4a). These relationships can be among entities like samples, cells, or spatial spots (entity-level), or among biological features like genes, proteins, or methylation sites (feature-level). They help capture structure and patterns within a single molecular modality without introducing the complexity of integrating across layers.

Intra-omics graphs are constructed from data within a single omics layer, where edges represent statistical or functional relationships among entities of the same type. These edges are typically defined using measures such as mutual information, correlation, or distance-based similarity, capturing shared trends or dependencies within the data. Nodes can represent either entities (e.g. samples, cells) or features (e.g. genes, proteins). At the entity level, samples can be connected based on similarities in molecular profiles within one omics layer. For example, MOGONET [64], MoGCN [69], SUPREME [71], and many others construct separate omics-specific graphs for layers such as mRNA, miRNA, or DNA methylation. At the feature level, intra-omics graphs link features showing coordinated activity across samples, reflecting potential regulatory or functional relationships. For example, TEMINET [81] derives intra-omics feature connections purely from correlation-based data-driven inference, while MPK-GNN [75] encodes gene-level interactions within a single omics by incorporating external gene interaction knowledge.

Due to their simplicity and expressive power, intra-omics graphs are widely used as an initial step in multi-omics analysis pipelines. Many frameworks first model within-layer relationships and then introduce cross-layer links through fusion modules [64, 71, 72]. However, because they represent only a single molecular layer, intra-omics graphs provide a limited perspective and can miss important cross-omics interactions. While valuable on their own, they are most effective when paired with inter-omics modeling to reveal the full complexity of biological systems.

#### Inter-omics graphs

Inter-omics graphs capture relationships across multiple omics layers, modeling how molecular features in one layer influence those in another (Fig. 4b). They reveal cross-omics dependencies essential for understanding complex biological regulation. For instance, miRNA-mRNA graphs represent post-transcriptional suppression, gene–protein graphs capture transcription-to-translation relationships, and methylation-gene expression graphs capture associations in which promoter methylation is often linked to reduced transcriptional activity. By encoding such inter-layer connections, inter-omics graphs provide a more comprehensive molecular view than intra-omics graphs alone.

Inter-omics graphs are mostly feature-level graphs because they allow for a direct modeling of interactions between molecular features from different omics layers. Nodes represent features from different omics, and edges denote biologically meaningful relationships. Construction may often rely on external biological knowledge as they can provide more reliable inter-omics connections. Structurally, inter-omics graphs may be bipartite or multipartite, linking features from different omics layers. For example, omicsGAN [70] and PTNet [65] use a miRNA–mRNA bipartite graph, whereas GLUE [93] connects genes to chromatin peaks and genes to methylation sites in a multipartite manner.

Inter-omics graphs can also be constructed in a data-driven manner by estimating cross-modality associations using similarity or correlation measures [146], or by combining separate omics-specific graphs with graph fusion approaches such as SNF [147]. Examples include MoGCN [69], DeepMoIC [77], and MOSEGCN [80]. However, directly inferring feature-to-feature cross-omics links from raw data is challenging due to missing values, small sample sizes, and inconsistent measurement scales. Meanwhile, knowledge-based construction requires harmonizing fragmented databases, resolving identifier mismatches, and accounting for tissue- or context-specific interactions.

In practice, inter-omics graphs are often used alongside intra-omics graphs. Models may learn intra-omics representations first and then refine or align them using inter-omics relationships. This hybrid design improves biological interpretability and robustness, as intra-omics structures (e.g. gene co-expression or PPI networks) remain essential foundations for meaningful cross-omics integration.

#### Mixed interaction graphs

Mixed interaction graphs combine intra-omics and inter-omics interaction edges within a single structure, allowing models to exploit both modality-specific neighborhoods and cross-layer couplings (Fig. 4c). Nodes such as genes, chromatin regions, proteins, cells, samples, or spatial spots retain within-layer links (e.g. gene–gene edges from co-expression or pathway membership, peak–peak edges from co-accessibility), while additional edges connect different layers, such as peak–gene regulatory links, gene–protein mappings, or cross-modality cell anchors. In this sense, ”mixed” refers to coexisting edge sources: multiple within-omics interaction types can be used alongside multiple cross-omics coupling types, all contributing to message passing paths that traverse within and across layers. An alternative mixed design keeps a single node type (often cells, spots or samples) but assigns multi-omics attributes to each node (e.g. RNA, ATAC, protein features), so the node simultaneously contains within-modality signals and cross-modality context; inter-omics coupling is then expressed through feature fusion/encoders and/or cross-modality consistency constraints, rather than through explicit cross-type edges.

In practice, a common recipe is to first construct intra-omics graphs that capture within-layer neighborhoods (from data-driven associations or curated interactions), and then introduce inter-omics edges using known mappings or anchor links so information can flow both within each layer and across layers. A complementary mixed design keeps a single node set (e.g. cells or spots) but attaches multi-omics feature channels to each node, coupling modalities through joint encoders or cross-modal consistency losses rather than explicit cross-type edges. AMOGEL [85] exemplifies the edge-mixing construction: it applies association rule mining to early-fused miRNA, mRNA, and DNA methylation to derive an information-based graph that captures both intra- and inter-omics associations, and then augments it with curated biological edges from resources such as STRING (PPI), KEGG, and GO to yield multi-dimensional connectivity. As an explicit graph-based example of anchor-driven coupling, GCN-SC [94] constructs a mixed cell graph that combines within-dataset neighborhood edges with cross-dataset anchor edges (MNN cell pairs), enabling information transfer across aligned cellular states. Because mixed constructions can become dense and involve multiple interacting edge sets, careful thresholding, weighting, and regularization are typically needed to keep the resulting graph tractable and stable.

### Integration strategy

The ultimate goal of multi-omics integration frameworks is to generate a meaningful joint representation that supports various downstream or upstream tasks. This integration can occur at different stages of the analytical pipeline, at the beginning, in the middle, or at the end, leading to three categories of integration strategies: early integration, intermediate integration, and late integration. These approaches differ in when and how data from multiple omics layers are combined. From a graph-centric standpoint, early integration fuses raw or preprocessed omics before any graph is built, yielding one unified feature space that drives a single graph construction. Intermediate integration first constructs omics-specific graphs and performs graph computations within each, then merges the resulting node/edge embeddings or latent spaces during modeling. Late integration keeps graphs and models separate per omics, produces independent embeddings or predictions, and combines them only at the decision stage, thus shifting fusion from pre-graph, to in-graph, to post-graph/prediction levels.

#### Early integration

**Figure 5:**
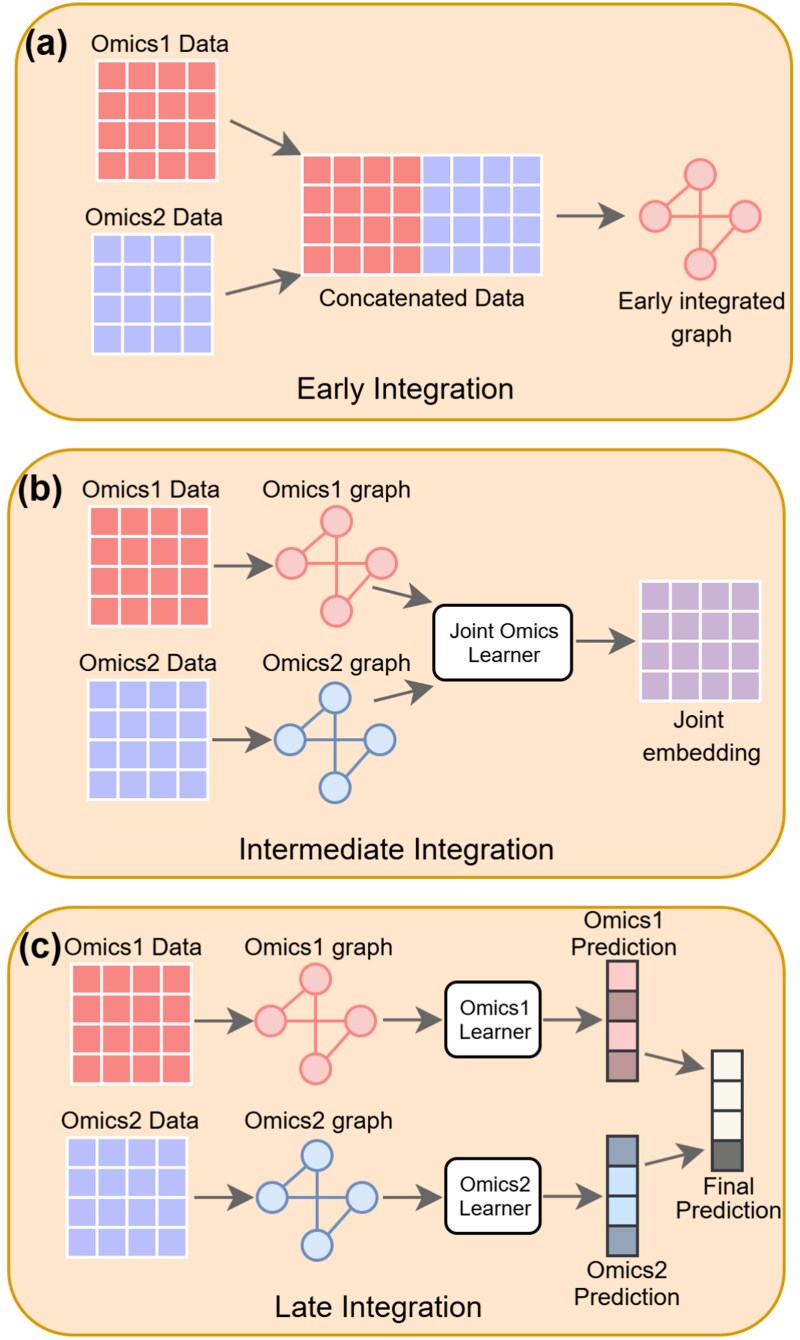
Omics integration strategies. (a) Early: omics data are concatenated and a single graph is built. (b) Intermediate: per-omics graphs are constructed and fused by a joint learner. (c) Late: omics-specific predictions are produced and their outputs are combined for the final prediction.

Early integration, often called data-level integration, is a straightforward, intuitive way to combine multi-omics data. Here, the different omics matrices are concatenated or otherwise merged into a single input representation before any modeling or graph construction occurs (Fig. 5a). A single model is then trained on this unified feature space, sharing parameters across modalities, treating all features uniformly at the input stage, and enabling the use of standard machine-learning or statistical techniques on the combined dataset.

Early integration is appealing due to its simplicity and its ability to expose models to a broad range of features across multiple modalities, which can reveal global cross-omics patterns that individual analyses might miss [148]. Many frameworks adopt this data integration approach. For example, MoGCN [69] uses an autoencoder to learn a shared feature space and constructs a combined graph, all based on the input omics matrices. EMOGI [66] concatenates all omics features into node attributes for its graph, which is built from external PPI networks. Similarly, DeepMAPS [97] first merges the modalities into a single-cell-gene activity matrix, and then constructs a single heterogeneous graph. However, concatenating different omics data types can obscure the unique distributions of each modality, which might lead to the introduction of non-biological artifacts [149]. The composite matrix generated in this way is often high-dimensional and uneven, increasing the risk of noise, overfitting, and slower learning. Additionally, differences in dataset sizes can create learning imbalances, where the model focuses more on omics with a higher number of features, neglecting others [150]. This approach is also sensitive to missing data and can hinder interpretability by blending the individual contribution of each modality. To mitigate these issues, early integration workflows typically first reduce complexity, usually by applying feature selection or dimensionality reduction, before passing the unified data to downstream models.

#### Intermediate integration

Intermediate integration, also referred to as model-level or joint integration, fuses modalities during modeling: each omics layer first forms its own graph and is processed to produce modality-specific embeddings. The method then jointly integrates these embeddings to learn both a common representation across omics and omics-specific representations that preserve topology and edge semantics [150]. This supports joint training with modality-specific parameters in early layers and shared parameters in fusion/prediction heads, while naturally reducing dimensionality and complexity (Fig. 5b).

This paradigm assumes that the omics can be projected into a shared latent space that captures common biological factors across all modalities. Each modality is first normalized or encoded to resolve scale and distribution mismatches, then fused in feature or latent space to model cross-omics interactions while preserving modality-specific signals. Graph-based formulations are often a good fit because they make cross-modal relationships explicit and can incorporate known correspondences (e.g. anchors or mappings) during fusion; many frameworks also support partial fusion or imputation-style training when some modalities are missing [148]. Compressing each omics layer beforehand also reduces computation and often improves performance. For example, omicsGAN [70] uses paired miRNA–mRNA profiles together with a bipartite regulatory network, applying iterative cross-updates to generate fused representations before prediction. Similarly, scMIC [96] learns modality-specific embeddings using graph autoencoders (GAEs) and then fuses them through latent-space redundancy reduction and cross-omics collaborative clustering. Both approaches exemplify intermediate integration.

Designing effective modality-specific encoders and deciding how and when to fuse the learned representations require careful consideration and domain knowledge. Poorly chosen integration strategies may lead to information loss or suboptimal performance. Additionally, training joint models can be computationally intensive and may require large, well-annotated datasets to avoid overfitting and ensure generalizability.

#### Late integration

Late integration, also known as decision-level or result-level integration, is a strategy where each omics modality is analyzed independently through separate models or pipelines, each with its own set of parameters, and the results are subsequently combined to form a final decision or prediction. Unlike early or intermediate integration, this approach does not attempt to merge raw data or intermediate features. Instead, each omics layer contributes to the final outcome through its own dedicated analysis, and the integration occurs only at the level of model output, such as prediction scores, cluster assignments, or inferred labels (Fig. 5(c)).

Late integration offers high flexibility and modularity [50]. Each modality is processed and modeled independently, with its own preprocessing, architectures, and hyperparameters, making it well suited to highly heterogeneous inputs, and naturally resilient to missing modalities. Because outputs remain modality-specific until the end, researchers can audit which omics contribute to the final decision, making the strategy interpretable. Fusion occurs at the decision stage, using tools such as voting or averaging schemes, ensemble learning, meta-classifiers, consensus clustering, or statistical decision rules. These methods combine per-omics predictions or embeddings without merging raw features or latent spaces, preserving modality transparency while still capturing complementary signals. In practice, MOGONET [64] trains omics-specific graph convolutional networks (GCNs) to produce class-probability vectors and then fuses them in label space via a view correlation discovery network, which models cross-omics correlations among the predicted labels. M$^{2}$CGCN [84] employs late integration by learning separate GCN-based cluster assignment distributions for each omics and then averaging these omics-specific soft labels across modalities. The final subtype for each sample is chosen from this averaged label distribution.

Late integration’s main drawback is its disjointed learning: modalities are modeled separately and fused only at the end, which can miss synergistic cross-omics interactions and hurt performance when signals are distributed across layers [150]. It also shifts complexity to the fusion design, where crafting an effective decision-level combiner is nontrivial and may itself introduce new failure modes.

### Graph context

Graph context describes the source of relational structure that a graph is intended to encode. This axis is especially important for spatial multi-omics, where graphs may represent either molecular relationships derived from omics measurements or physical neighborhood relationships derived from tissue coordinates. We distinguish two broad graph contexts: molecular graphs and spatial graphs. Molecular graphs encode relationships defined by molecular profiles, molecular features, or biological prior knowledge, whereas spatial graphs encode the physical organization of cells or spots in tissue.

#### Molecular graph

We use the term molecular graph to refer broadly to graphs whose topology is derived from omics measurements or molecular biological knowledge, rather than from physical tissue coordinates. This includes entity-level graphs where samples, cells, or spots are connected based on molecular-profile similarity, feature-level graphs where genes, proteins, peaks, methylation sites, or metabolites are linked by regulatory, functional, or statistical relationships, and entity–feature graphs where observed molecular measurements define edges between entities and features. Thus, most graph categories discussed in the previous axes, including data-driven, knowledge-based, hybrid, intra-omics, inter-omics, and mixed graphs, can be interpreted as molecular graphs when their edges are defined from molecular information.

Molecular graphs are useful for modeling biological similarity, regulatory dependencies, pathway structure, and cross-omics interactions. In bulk and single-cell integration, graph construction is generally based on molecular features, because these settings do not provide an explicit tissue coordinate system. Accordingly, all surveyed bulk and single-cell methods fall under the molecular graph category. In spatial multi-omics, molecular graphs provide information that is complementary to spatial adjacency, allowing models to capture molecular similarities or regulatory couplings that may not be explained by physical proximity alone. Several surveyed spatial methods, including SpatialGLUE [111], SSGATE [112], and SEPAR [126], therefore use molecular graphs in addition to spatial graphs.

#### Spatial graph

Spatial graphs encode physical neighborhood structure in tissue. In these graphs, nodes usually represent spots or cells, and edges are defined using spatial coordinates, capture-array layout, distance thresholds, or nearest-neighbor relationships in physical space. Unlike molecular graphs, spatial graphs do not necessarily encode molecular similarity or regulatory relationships. Instead, they model the assumption that nearby cells or spots are likely to share tissue context, microenvironmental influence, or spatially coordinated biological states.

This distinction is important because a spatial graph can exist even when omics interactions are not represented as graph relations. For example, a method may connect neighboring spots or cells using tissue coordinates while using molecular measurements only as node attributes. In such cases, the graph supports spatial smoothing, spatial-domain identification, or tissue-structure-aware representation learning, but the edges themselves do not encode intra-omics or inter-omics molecular relationships. Nearly all surveyed spatial methods use spatial information to construct graph structure in some form. Methods, such as soFusion [117], COSMOS [121], and spaMGCN [122], rely only on spatial graphs without explicitly constructing molecular graphs.

In spatial multi-omics models, it is also common to use both spatial and molecular graphs. Some methods merge spatial and molecular edges into a unified graph, whereas others process the two graph types through separate modules before fusing their embeddings. Methods such as SWITCH [119], spaLLM [118], and SpaBalance [114] construct both spatial and molecular graph structures to capture complementary tissue-neighborhood and molecular relationships. MISO [120] is the only exception among the surveyed spatial methods: it uses a molecular graph as the graph structure in its pipeline, while incorporating spatial coordinates as feature attributes rather than as spatial adjacency edges. Thus, graph context specifies the source of structural information used by each spatial method, whether tissue geometry, molecular relationships, or both.

### Graph-based deep learning architectures

Graph-based deep learning models provide a natural and expressive framework for multi-omics integration because they represent biological systems as networks of interacting molecular components. Biological data rarely exist in isolation. Genes regulate other genes through their encoded regulatory proteins or RNAs, proteins form extensive interaction networks, and epigenetic modifications influence both nearby and distal genomic regions. Together, these processes give rise to complex dependency structures within and across omics layers. GNNs are particularly well-suited for this context because they model such relational dependencies directly, using graph structures where nodes represent biological entities and edges encode functional, physical, or statistical relationships. This allows GNNs to capture localized and higher-order neighborhood effects that are critical in understanding regulatory cascades or disease mechanisms. Beyond GNNs, advanced deep learning architectures such as autoencoders (AEs), variational autoencoders (VAEs), transformers and generative adversarial networks (GANs) are increasingly being adapted to graph-structured data, typically by incorporating graph-aware inductive biases (e.g. neighborhood aggregation or diffusion) into their encoder–decoder components. These models combine the expressive power of latent-variable frameworks with graph-based learning, enabling more flexible and biologically grounded integration strategies. As a result, the field is moving beyond flat, feature-level fusion toward graph-aware, structure-preserving integration that more accurately reflects the multi-layered complexity of biological systems.

#### Graph convolutional networks

GCNs [151] have become one of the foundational tools in graph-based multi-omics integration due to their capacity to capture local neighborhood structures and propagate information across graph nodes. Given an adjacency $\tilde{A}$ with self-loops and degree matrix $\tilde{D}$, a $(l+1)^{\textrm{th}}$ GCN layer updates node features $X$ via: 


\begin{align*}& X^{(l+1)} = \sigma\left( \tilde{D}^{-1/2} \tilde{A} \tilde{D}^{-1/2} X^{(l)} W^{(l)} \right), \end{align*}

learning topology-aware embeddings from the graph.

In multi-omics settings, $\tilde{A}$ may encode sample similarity, cell–cell similarity, feature similarity, spatial proximity, or prior biological relations. Stacking GCN layers aggregates information from progressively larger neighborhoods, allowing the learned representations to capture both local interactions and the broader topology of the network. However, the biological meaning of the learned representation depends strongly on how the graph is constructed and how modality-specific embeddings are integrated.

Representative methods illustrate different uses of GCNs across bulk, single-cell, and spatial multi-omics. MOGONET [64] applies GCNs to supervised bulk multi-omics classification by constructing a separate patient similarity graph for each omics modality, such as mRNA, DNA methylation, or miRNA. Each omics-specific GCN learns class predictions by combining patient-level molecular features with patient-patient similarity, and the modality-specific predictions are subsequently fused to capture complementary class-level signals across omics layers. In scGCN [91], the graph is constructed over cells rather than patients. Reference and query cells are connected through a mixed interaction graph that captures both intra-dataset and inter-dataset cell mappings, and a semi-supervised GCN propagates label information from annotated reference cells to unlabeled query cells. SpatialGLUE [111] adapts GCNs to spatial multi-omics by constructing, within each modality, both a spatial graph from tissue coordinates and a feature graph from molecular similarity. Separate GCN encoders learn graph-specific representations, which are then combined through within-modality and between-modality attention. Overall, GCNs can function either as simple standalone graph encoders or as the backbone of more complex multi-omics integration models.

#### Graph attention networks

Graph attention networks (GATs) [152] extend the capability of GCNs by incorporating attention mechanisms that allow the model to assign different weights to neighboring nodes during the aggregation process. This is especially beneficial in multi-omics data where not all connections are equally informative or biologically relevant. The attention coefficient $\alpha $ between nodes $i$ and $j$ is computed as: 


\begin{align*}& \alpha_{ij} = \frac{\exp\left(\textrm{LeakyReLU}\left(a^\top [W h_{i} \, \| \, W h_{j}]\right)\right)}{\sum_{k \in \mathcal{N}(i)} \exp\left(\textrm{LeakyReLU}\left(a^\top [W h_{i} \, \| \, W h_{k}]\right)\right)}, \end{align*}

and the new node representation is: 


\begin{align*}& h_{i}^{\prime} = \sigma\left( \sum_{j \in \mathcal{N}(i)} \alpha_{ij} W h_{j} \right) \end{align*}

GATs replace uniform neighbor aggregation with learned, context-dependent attention weights, allowing each node to prioritize more informative neighbors during message passing. This is useful in multi-omics graphs where edges may reflect noisy sample similarity, molecular associations, spatial proximity, or heterogeneous gene relationships. In MOGAT [76], patients are treated as nodes in omics-specific patient similarity networks, and GATs generate embeddings for cancer subtype prediction, visualization, and survival analysis. In MODIG [68], genes are nodes in a multi-dimensional gene network, with edges derived from protein–protein interaction, co-expression, pathway, sequence, and Gene Ontology relationships; GAT blocks learn dimension-specific gene representations that are fused for cancer driver gene prediction. In SSGATE [112], graph attention auto-encoders are used in a dual-path framework for transcriptome and proteome integration, where neighborhood graphs are constructed from single-cell expression profiles or spatial coordinates, and the learned modality-specific embeddings are combined into a joint representation for downstream analysis. Thus, GATs provide an adaptive graph encoding strategy that can be used either directly or as part of larger multi-omics integration frameworks.

#### Graph autoencoders and variational graph autoencoders

Autoencoders (AEs) and variational autoencoders (VAEs) [153, 154], when extended to graph structures, serve as powerful tools for integrating multi-omics data by learning latent embeddings that capture both molecular variation and topological structure. Instead of treating omics layers as flat matrices, these models rely on biological graphs to guide how the data are encoded. This enables the latent space to reflect functional relationships, not just statistical co-variation.

In GAEs, a graph encoder learns node embeddings from features and topology, and a decoder reconstructs the graph: 


\begin{align*}& Z = \mathrm{GCN}(X,A), \qquad \hat{A} = \sigma(ZZ^\top) \end{align*}

VGAEs introduce a probabilistic latent space by estimating $\mu $ and $\log \sigma ^{2}$ with GCN encoders and sampling embeddings through the reparameterization trick: 


\begin{align*}& \mu = \mathrm{GCN}_{\mu}(X,A), \quad \log\sigma^{2} = \mathrm{GCN}_{\log\sigma^{2}}(X,A), \end{align*}

\begin{align*}& Z = \mu + \sigma \odot \epsilon,\;\; \epsilon \sim \mathcal{N}(0,I) \end{align*}

The VGAE objective balances adjacency reconstruction with regularization toward a prior distribution: 


\begin{align*}& \mathcal{L}_{\mathrm{VGAE}} = \mathbb{E}_{q(Z|X,A)}[\log p(A|Z)] - \mathrm{KL}\!\left(q(Z|X,A)\,\|\,p(Z)\right). \end{align*}

In GAE-based models, the goal is to learn compact latent representations from graph-structured omics data and then reconstruct either the original features, graph structure, or both. VGAEs extend this idea by learning distributions over the latent space, which can improve robustness and support generative modeling. These architectures are especially useful in unsupervised or weakly supervised settings, where informative embeddings must be learned without relying on extensive labels. In MoGCN [69], an AE is first used to reduce the dimensionality of multi-omics patient profiles and extract latent patient features, while similarity network fusion constructs a patient similarity network; the learned features and graph structure are then passed to a GCN for cancer subtype classification. In GLUE [93], omics-specific VAEs learn cell embeddings from unpaired single-cell modalities, while a VGAE learns feature embeddings from a prior knowledge-based guidance graph linking regulatory features across omics layers. These feature embeddings help align distinct modality-specific feature spaces and enable both cell-state integration and regulatory inference. In SSGATE [112], graph attention AEs are arranged in a dual-path framework for transcriptome and proteome integration. Each path encodes one modality together with its neighborhood graph, which is built from single-cell expression profiles or spatial coordinates, and the resulting embeddings are combined into a joint representation while reconstruction losses preserve modality-specific information. However, AEs can suffer from limited regularization and produce overly compact embeddings, whereas VAEs, although generally more stable, can smooth features too aggressively unless their training is carefully balanced.

#### Generative adversarial networks

Graph-based generative adversarial networks (GANs) and adversarial training strategies have emerged as powerful tools for modeling omics data with high variability and complex distributions. In these setups, a generator network aims to synthesize realistic omics profiles or latent embeddings, while a discriminator distinguishes between real and generated samples. When graphs are involved, the generator often produces latent node embeddings conditioned on graph structure, while the discriminator assesses the authenticity of embeddings or reconstructed graphs.

A standard GAN consists of a generator $G$ that maps noise $Z$ to a generated sample $\hat{X}$, and a discriminator $D$ that estimates whether a sample is real: 


\begin{align*}& \hat{X} = G(Z), \quad Z \sim p_{Z}(Z), \qquad D(X) \in (0,1). \end{align*}

The generator and discriminator are trained through the minimax objective: 


\begin{align*}& \min_{G} \max_{D} \; \mathbb{E}_{X\sim p_{\mathrm{data}}}[\log D(X)] + \mathbb{E}_{Z\sim p_{Z}}[\log(1-D(G(Z)))]. \end{align*}

For multi-omics integration, adversarial models can be used to align or enrich modality-specific representations by forcing generated profiles to resemble real omics data while retaining information transferred from another modality. This is useful for cross-omics representation learning, synthetic profile generation, and improving downstream prediction when omics layers are linked by known biological interactions. In omicsGAN [70], mRNA and miRNA expression profiles are integrated together with a bipartite miRNA–mRNA regulatory network. The model uses paired Wasserstein GAN modules to iteratively update each omics profile: one generator produces a synthetic representation of one omics layer using the other omics layer and the normalized interaction matrix, while a critic encourages the generated profile to match the distribution of the corresponding real omics data. The resulting synthetic omics profiles are then used for cancer phenotype classification and survival prediction. This design shows how adversarial learning can incorporate inter-omics regulatory structure rather than simply concatenating modalities. However, GAN-based integration can be sensitive to hyperparameters, training stability, and interaction-network quality, and generated profiles may be difficult to interpret if the biological constraints are weak or incomplete.

#### Transformers

g While originally developed for sequence modeling, transformers [155] have recently been adapted for graph-based omics integration through graph-transformer hybrids [156]. These models replace or augment standard graph convolution operations with self-attention mechanisms, allowing the model to learn global dependencies across nodes irrespective of graph locality. In biological networks where long-range dependencies, such as those between regulatory genes and distal targets, play an important role, transformers provide a powerful modeling tool.

Given input $X \in \mathbb{R}^{n \times d}$, a transformer layer computes: 


\begin{align*}& Q = X W_{Q},\quad K = X W_{K},\quad V = X W_{V}, \end{align*}

\begin{align*}& \textrm{Attention}(Q,K,V) = \operatorname{softmax} \left(\frac{QK^\top}{\sqrt{d_{k}}}\right)V, \end{align*}

\begin{align*}& X^{\prime} = X + \textrm{Attention}(Q,K,V), \quad Y = X^{\prime} + \operatorname{FFN}(X^{\prime}), \end{align*}

where $Y$ is the layer output and $\operatorname{FFN}(x) = \sigma (x W_{1} + b_{1}) W_{2} + b_{2}$, optionally combined with layer normalization at each residual step.

Transformer-based models use self-attention to learn dependencies among omics features, samples, or graph nodes after projecting them into a shared embedding space. This allows the model to capture long-range cross-omics relationships without requiring every interaction to be specified as an explicit graph edge, although graph structure can still be incorporated through learned sample networks, heterogeneous graphs, or attention-guided message passing. In MOGLAM [72], omics-specific dynamic GCNs first learn adaptive sample-level embeddings, and a transformer-inspired multi-head attention module is then used to capture common and complementary information across omics for patient classification. In MOSEGCN [80], a transformer encoding module learns latent feature correlations within and across omics, while similarity network fusion constructs a patient similarity graph that is passed to a semi-supervised GCN classifier. In MarsGT [99], a heterogeneous graph transformer jointly models cells, genes, and peaks from single-cell multi-omics data, using attention-based message passing and probability-based subgraph sampling to identify rare cell populations and infer regulatory relations. These examples show that transformer-style attention can support flexible cross-omics integration, but the models can be computationally demanding and may require careful regularization to avoid unstable or difficult-to-interpret associations.

#### Others

Beyond the commonly used GCN, GAT, GAE, VGAE, GAN, and transformer-based architectures, several graph-based multi-omics methods employ alternative or extended designs. GraphSAGE is useful for neighborhood aggregation and inductive message passing; in scMoFormer [95], it is used to transfer information across gene–protein and gene–cell links, thereby connecting modality-specific transformers through the heterogeneous graph. Hypergraph neural networks generalize pairwise message passing by connecting multiple related nodes through a single hyperedge, enabling higher-order relationships among cells or modalities. For example, scHyper [101] constructs modality-specific cell hypergraphs for RNA–ATAC integration, while scMHNN [106] combines RNA, ATAC, and protein hyperedges into a unified multi-omics hypergraph for tri-modal representation learning. We classify these methods as homogeneous and entity-level because all hypergraph vertices represent the same biological entity type, i.e. cells. They are also categorized as intra-omics because the hyperedges are constructed within each modality-specific cell space, and cross-omics information is integrated by combining modality-specific hyperedges rather than by explicitly defining molecular cross-omics edges. Some methods use neural networks with graph-derived inputs rather than explicit GNN layers: network-based fusion [67] constructs patient similarity networks, extracts centrality and modularity features, and feeds these graph features into neural or machine-learning classifiers, while MinNet [110] uses a Siamese neural network trained with a graph-based contrastive loss in which a k-NN graph defines cell-pair distances. Other methods rely on matrix factorization modules, such as SEPAR [126], which applies graph-regularized NMF to decompose spatial omics data into metagene patterns while using a spatial graph Laplacian to preserve neighborhood structure. These examples show that graph-based multi-omics models are not limited to canonical GNN layers, since graph information can also guide sampling, loss design, feature extraction, matrix factorization, or communication between broader neural modules.

A concise comparison of major graph design choices, along with their representative methods and practical implications, is provided in Table 1.

**Table 1:** Trade-offs of major graph design choices in graph-based multi-omics integration. The table summarizes representative methods, advantages, limitations, and suitable application settings for common node schema, edge semantics, integration strategy, and graph context choices

**Design axis**	**Design choice**	**Typical methods**	**Advantages**	**Limitations**	**Best suited for**
**Node schema**	Entity-level graph	MOGONET, scGCN, SpatialGLUE	Directly supports sample/cell/spot prediction; easy supervision; scalable	Less molecular interpretability; similarity sensitive to noise	Classification, clustering, domain detection
	Feature-level graph	EMOGI, GLUE, GOAT	Better mechanistic interpretation; supports regulatory/pathway reasoning	Requires reliable priors or enough samples; harder to link to labels	Biomarker discovery, regulatory inference
	Entity–feature graph	scMoGNN, DeepMAPS	Joint entity and feature embeddings; supports attribution	Large sparse graph; sensitive to measurement noise	Single-cell multimodal prediction, marker discovery
**Edge semantics**	Data-driven edges	MOGONET, scGCN, SpaMGCN	Flexible; no external database required	Sensitive to preprocessing, batch, k-NN choice	Cohort-specific discovery
	Knowledge-based edges	EMOGI, PTNet, GLUE	Interpretable; biologically grounded	Incomplete, biased, static priors	Mechanistic modeling
	Hybrid edges	AMOGEL, scMoGNN, MultiGATE	Balances prior knowledge and data adaptation	More hyperparameters; harder to validate	Noisy or low-sample settings
**Integration**	Early integration	MoGCN, AMOGEL	Simple; captures global signal	Modality imbalance; missing data issues	Well-normalized complete data
	Intermediate integration	GLUE, scMIC, SpatialGLUE	Balances modality-specific and joint learning	More complex; needs careful tuning	Most multi-omics settings
	Late integration	MOGONET, SUPREME	Modular; robust to missing modalities	May miss cross-omics synergy	Clinical prediction with incomplete modalities
**Graph context**	Molecular graph	GLUE, MISO, MultiGATE	Captures molecular similarity, regulation, or cross-omics relationships	May miss physical tissue organization	Molecular programs, regulatory inference
	Spatial graph	COSMOS, spaMGCN, spaMI, SpatialGLUE	Captures tissue neighborhood structure	Does not necessarily encode molecular interactions	Spatial domain detection, tissue architecture

**Table 2:** Graph-based deep learning methods for bulk multi-omics integration, summarizing the omics used (DM: DNA methylation, CNV: copy-number variation, GV: genomic variants, HM: histone modifications, PROT: proteome, MET: metabolome, CoExp: co-expression network, Clin: clinical/phenotypic; transcriptomic inputs are listed explicitly as mRNA, miRNA and lncRNA), node schema, edge semantics, omics interaction type, integration strategy, and model architecture. In the node schema column, “homo” denotes homogeneous, “hetero” denotes heterogeneous, EE denotes entity-level, FF denotes feature-level, and EF denotes entity-feature graphs

**Model**	**Omics used**	**Node schema**	**Edge semantics**	**Omics interaction**	**Integration strategy**	**Architecture**
MOGONET [64]	mRNA, miRNA, DM	Homo, EE	Data-driven	Intra-omics	Late	GCN
PTNET [65]	mRNA, miRNA	Hetero, FF	Knowledge-based	Inter-omics	Intermediate	NN
EMOGI [66]	GV, CNV, mRNA, DM	Homo, FF	Knowledge-based	Mixed	Early	GCN
Wang *et al*. [67]	mRNA, DM	Homo, EE	Data-driven	Intra-omics	Intermediate	NN
MODIG [68]	GV, CNV, GE, DM	Homo, FF	Hybrid	Mixed	Early	GAT
MoGCN [69]	CNV, mRNA, PROT	Homo, EE	Data-driven	Intra-omics	Early	GCN, AE
omicsGAN [70]	mRNA, miRNA	Hetero, FF	Knowledge-based	Inter-omics	Intermediate	GAN
SUPREME [71]	mRNA, DM, miRNA, CNV, CoExp, GV, Clin	Homo, EE	Data-driven	Intra-omics	Late	GCN
MOGLAM [72]	mRNA, miRNA, DM	Homo, EE	Data-driven	Intra-omics	Intermediate	GCN Transformer
MRGCN [73]	mRNA, miRNA, DM, CNV	Homo, EE	Data-driven	Intra-omics	Intermediate	GCN
GOAT [74]	mRNA, DM, PROT, MET	Homo, FF	Knowledge-based	Mixed	Early	GAT
MPK-GNN [75]	mRNA, CNV	Homo, FF	Knowledge-based	Mixed	Early	GCN
MOGAT [76]	mRNA, miRNA, lncRNA, DM, CNV, GV, CoExp, Clin	Homo, EE	Data-driven	Mixed	Early	GAT
DeepMoIC [77]	mRNA, CNV, DM, miRNA, PROT	Homo, EE	Data-driven	Intra-omics	Intermediate	GCN, AE
Yan *et al*. [78]	mRNA, CNV, DM	Hetero, FF	Knowledge-based	Mixed	Intermediate	GraphSAGE
Subtype-MGTP [79]	mRNA, miRNA, CNV, DM PROT	Homo, EE	Data-driven	Intra-omics	Intermediate	GCN
MOSEGCN [80]	mRNA, miRNA, DM	Homo, EE	Data-driven	Intra-omics	Early	Transformer AE, GCN
TEMINET [81]	mRNA, miRNA, DM	Homo, FF	Data-driven	Intra-omics	Late	GAT, NN
MCRGCN [82]	mRNA, miRNA, DM	Homo, EE	Data-driven	Intra-omics	Intermediate	GCN
GREMI [83]	mRNA, miRNA, DM	Homo, FF	Data-driven	Intra-omics	Intermediate	GAT
M$^{2}$CGCN [84]	mRNA, miRNA, DM, CNV	Homo, EE	Data-driven	Intra-omics	Late	GCN, MLP
AMOGEL [85]	mRNA, miRNA, DM	Hetero, FF	Hybrid	Mixed	Early	GAT
GNNRAI [86]	mRNA, PROT	Homo, FF	Knowledge-based	Intra-omics	Intermediate	GCN Transformer
mmMOI [87]	mRNA, miRNA, DM	Homo, EE	Data-driven	Intra-omics	Intermediate	GCN, AE
SynOmics [88]	mRNA, miRNA, DM	Homo, FF Hetero, FF	Hybrid	Intra-omics Inter-omics	Intermediate	GCN
IGCN [89]	mRNA, miRNA, DM	Homo, EE	Data-driven	Intra-omics	Intermediate	GCN
EpGAT [90]	HM, Hi-C, mRNA	Homo, EE	Data-driven	Intra-omics	Early	GAT

**Table 3:** Graph-based deep learning methods for single-cell multi-omics integration, summarizing the omics used (mRNA, ATAC: chromatin accessibility, DM: DNA methylation, PROT: proteome), node schema, edge semantics, omics interaction type, integration strategy, and model architecture. In the node schema column, “homo” denotes homogeneous, “hetero” denotes heterogeneous, EE denotes entity-level, FF denotes feature-level, and EF denotes entity-feature graphs

**Model**	**Omics used**	**Node schema**	**Edge semantics**	**Omics interaction**	**Integration strategy**	**Architecture**
scGCN [91]	mRNA, ATAC	Homo, EE	Data-driven	Mixed	Intermediate	GCN
scMoGNN [92]	mRNA, ATAC, PROT	Hetero, EF	Hybrid	Mixed	Intermediate	GCN
GLUE [93]	mRNA, ATAC, DM	Hetero, FF	Knowledge-based	Inter-omics	Intermediate	VGAE
GCN-SC [94]	mRNA, ATAC, PROT	Homo, EE	Data-driven	Mixed	Intermediate	GCN
scMoFormer [95]	mRNA, PROT	Hetero, EF	Hybrid	Mixed	Intermediate	Transformer GraphSAGE
scMIC [96]	mRNA, ATAC, PROT	Homo, EE	Data-driven	Intra-omics Inter-omics	Intermediate	GAE
DeepMAPS [97]	mRNA, ATAC, PROT	Hetero, EF	Data-driven	Mixed	Early	Transformer GAE
scCross [98]	mRNA, ATAC, DM, PROT	Homo, EE	Data-driven	Intra-omics	Intermediate	VAE, GCN GAN
MarsGT [99]	mRNA, ATAC	Hetero, EF	Hybrid	Inter-omics	Early	Transformer
scMFC [100]	mRNA, ATAC	Homo, EE	Data-driven	Intra-omics	Intermediate	Transformer
scHyper [101]	mRNA, ATAC, PROT	Homo, EE	Data-driven	Intra-omics	Intermediate	NN
scGDCC [102]	mRNA, ATAC, PROT	Homo, EE	Data-driven	Intra-omics	Intermediate	GAE
scMDCL [103]	mRNA, ATAC	Homo, EE	Data-driven	Intra-omics	Intermediate	GAE
scMID [104]	mRNA, ATAC	Homo, EE, FF	Data-driven	Intra-omics	Intermediate	GCN
scSPAF [105]	mRNA, ATAC	Homo, EE	Data-driven	Intra-omics	Intermediate	GCN, AE
scMHNN [106]	mRNA, ATAC, PROT	Homo, EE	Data-driven	Mixed	Early	GNN
scMGCL [107]	mRNA, ATAC	Homo, EE	Data-driven	Intra-omics	Intermediate	GCN
SuperGLUE [108]	mRNA, ATAC PROT, DM	Hetero, FF	Knowledge-based	Mixed	Intermediate	VAE, GCN GAN
scPairing [109]	mRNA, ATAC, PROT	Homo, EE	Data-driven	Inter-omics	Intermediate	VAE
MinNet [110]	mRNA, ATAC, PROT	Homo, EE	Data-driven	Intra-omics	Intermediate	NN

**Table 4:** Graph-based deep learning methods for spatial multi-omics integration, detailing the omics used (mRNA: transcriptome, PROT: proteome, ATAC: chromatin accessibility, HM: histone modifications, MET: metabolome), node schema, edge semantics, graph context, omics interaction type, integration strategy, and model architecture. In the node schema column, “homo” denotes homogeneous, “hetero” denotes heterogeneous, EE denotes entity-level, FF denotes feature-level, and EF denotes entity-feature graphs. A dash (–) entry under “Omics interaction” means omics interactions are not represented as graph relations—spatial adjacency alone is captured

**Model**	**Omics used**	**Node schema**	**Edge semantics**	**Graph context**	**Omics interaction**	**Integration strategy**	**Architecture**
SpatialGLUE [111]	mRNA, PROT, ATAC, HM	Homo, EE	Data-driven	Spatial molecular	Intra-omics	Intermediate	GCN
SSGATE [112]	mRNA, PROT	Homo, EE	Data-driven	Spatial molecular	Intra-omics	Intermediate	GAT, AE
SMMGCL [113]	mRNA, PROT, ATAC	Homo, EE	Data-driven	Spatial molecular	Mixed	Intermediate	GCN
SpaBalance [114]	mRNA, PROT, ATAC, HM	Homo, EE	Data-driven	Spatial molecular	Intra-omics	Intermediate	GCN
SpaOmicsVAE [115]	mRNA, PROT, ATAC, HM	Homo, EE	Data-driven	Spatial molecular	Intra-omics	Intermediate	GCN
MultiGATE [116]	mRNA, ATAC, PROT, MET	Homo, EE Hetero, FF	Hybrid	Spatial molecular	Mixed	Intermediate	GAT, AE
soFusion [117]	mRNA, PROT, ATAC, HM	Homo, EE	Data-driven	Spatial	–	Intermediate	GCN
spaLLM [118]	mRNA, ATAC, PROT	Homo, EE	Data-driven	Spatial molecular	Intra-omics	Intermediate	GCN
SWITCH [119]	mRNA, ATAC, HM	Homo, EE Hetero, FF	Hybrid	Spatial molecular	Mixed	Intermediate	GAT
MISO [120]	mRNA, ATAC, HM, PROT, MET	Homo, EE	Data-driven	Molecular	Intra-omics	Intermediate	NN
COSMOS [121]	mRNA, ATAC, PROT	Homo, EE	Data-driven	Spatial	–	Intermediate	GCN
spaMGCN [122]	mRNA, PROT, ATAC, HM	Homo, EE	Data-driven	Spatial	–	Intermediate	GCN
stClinic [123]	mRNA, ATAC, PROT	Homo, EE	Data-driven	Spatial molecular	Intra-omics	Intermediate	VGAE
spaMI [124]	mRNA, ATAC, PROT	Homo, EE	Data-driven	Spatial	–	Intermediate	GCN
SpatialEx [125]	mRNA, PROT, MET	Homo, EE	Data-driven	Spatial	–	Intermediate	HGNN
SEPAR [126]	mRNA, ATAC, PROT	Homo, EE	Data-driven	Spatial molecular	Intra-omics	Early	NMF

## Empirical design patterns across methods

**Figure 6:**
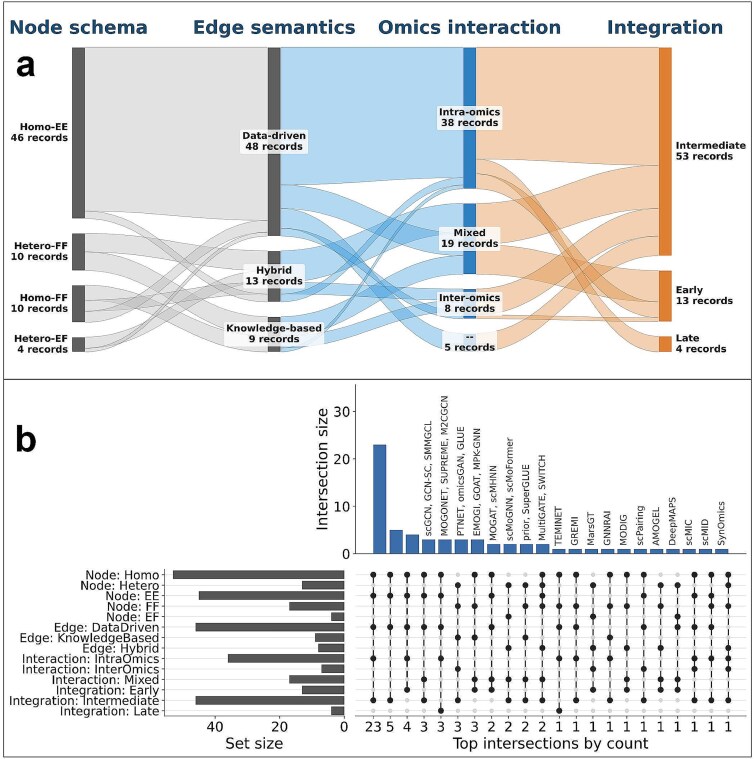
Empirical design patterns across graph-based multi-omics integration methods. (a) Sankey plot summarizing four shared design axes: node schema, edge semantics, omics interaction, and integration strategy, across 63 methods. (b) UpSet plot showing the most frequent intersections among the same shared design categories.

To complement the taxonomy with an empirical overview of the surveyed methods, we summarize 27 bulk, 20 single-cell, and 16 spatial multi-omics integration methods in Tables 2–4 respectively. We further highlight the recurring combinations of design choices across these methods in Fig. 6. This empirical summary emphasizes the design axes common to bulk, single-cell, and spatial methods, namely node schema, edge semantics, omics interaction, and integration strategy, while graph context is handled separately as a spatial-specific axis. Across the surveyed methods, the most common design is the homogeneous entity-level graph. As shown in Fig. 6a, this node schema is most frequently paired with data-driven edge construction and intra-omics interaction modeling. This pattern is common because entity-level graphs are straightforward to construct from available multi-omics datasets: patients, cells, or spots naturally serve as nodes, and edges can be defined using profile similarity, k-NN, or spatial adjacency without requiring curated molecular interaction databases. Such graphs also align well with common downstream tasks, such as patient classification, cell clustering, and spatial-domain identification, where predictions or embeddings are ultimately needed at the entity level. In addition, data-driven entity graphs are flexible across bulk, single-cell, and spatial settings, making them easier to adapt than feature-level or heterogeneous graphs that require explicit molecular priors or more complex graph construction. Figure 6b supports this observation by identifying the combination of homogeneous, entity-level, data-driven, intra-omics, and intermediate-integration choices as the largest recurring design pattern.

By contrast, feature-level methods, whether homogeneous or heterogeneous, more often rely on knowledge-based or hybrid edge semantics and are more frequently associated with inter-omics or mixed interaction structures. This pattern is expected because explicit cross-layer relations, such as miRNA–mRNA, gene–protein, methylation–gene, or pathway-based links, are naturally defined among molecular features rather than among biological entities. Heterogeneous entity–feature graphs show another distinct pattern: they are never purely knowledge-based, because entity–feature edges depend on observed measurements linking samples, cells, or spots to molecular features, and therefore must be derived from the data or combined with biological priors in a hybrid strategy. Intermediate integration is the most common fusion strategy, likely because it enables modality-specific representation learning while still supporting joint cross-omics modeling, providing a compromise between early concatenation and late decision-level fusion.

Spatial methods exhibit an additional design aspect that is absent in bulk and single-cell settings: whether the graph encodes spatial context, molecular context, or both. Most spatial methods use tissue coordinates to construct spatial graphs over cells or spots. Since these graphs are built at the cell/spot level, they are typically entity-level and homogeneous. Their edges usually come from tissue coordinates or spatial neighborhoods, which also makes them generally data-driven. Intra-omics labels are common, as spatial graphs typically connect neighboring spots or cells within a modality-specific spatial structure rather than explicitly encoding molecular links across omics layers. Some spatial methods also construct molecular graphs based on feature similarity, regulatory relationships, or other molecular associations. When both spatial and molecular graphs are used, they may be merged into a unified graph or processed by separate model components before integration. Most spatial methods use intermediate integration, where each modality-specific representation is encoded before fusion so that distinct tissue-level signals can be combined into spatially informed embeddings. GCN-based architectures are frequently used in this setting, providing a simple and effective backbone for propagating information through spatial neighborhoods.

Overall, these empirical patterns show that the proposed axes are useful for systematically annotating graph-based multi-omics methods, while also revealing that many design choices are coupled rather than fully independent. Recognizing these recurring patterns helps explain dominant design choices across data settings and can also highlight underexplored graph-design combinations for future method development.

## Graph-based multi-omics integration: a general pipeline

**Figure 7:**
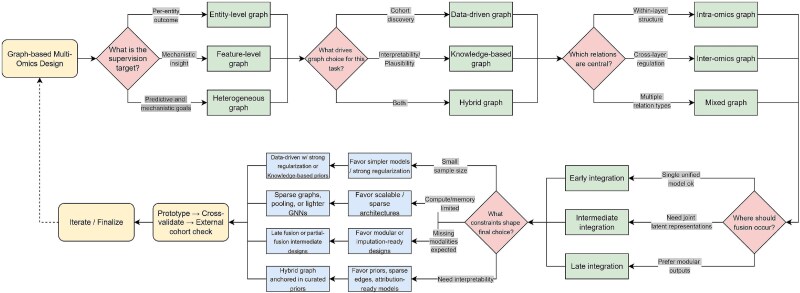
Decision flowchart for designing graph-based multi-omics integration pipelines, showing how supervision targets, graph construction choices, central relationship types, fusion stage, and practical constraints jointly guide model selection, prototyping, and iterative refinement.

A graph-based multi-omics pipeline is not a fixed architecture but a series of interdependent design decisions guided by the scientific objective and the data at hand. From selecting the appropriate node perspective to defining graph structure, modeling omics interactions, and choosing an integration strategy, each step balances discovery against interpretability and complexity against robustness. These decisions shape not only predictive performance but also biological insight, reproducibility, and generalizability. Understanding the rationale behind each stage is therefore essential for constructing graph models that are both effective and biologically meaningful. Figure 7 summarizes this decision process in a flowchart.

### Defining nodes

In graph-based multi-omics, the first decision is the view of the nodes (node schema), and it is best anchored to the scientific target. When outcomes attach to samples, cells, or spatial spots, an entity-level graph is usually selected so that each node maps directly to a prediction or label; diagnosis, prognosis, patient stratification, cell-type calling, trajectory inference, spatial domain segmentation, and spot deconvolution are typical examples. This choice simplifies supervision, loss design, and evaluation (e.g. Area Under the Receiver Operating Characteristic curve (AUROC) per patient or Adjusted Rand Index (ARI)/ Normalized Mutual Information (NMI) per cell cluster). A feature-level graph is often preferred when the goal is to uncover mechanisms such as pathway activity, regulatory programs, marker prioritization, target discovery, or link prediction, because it places relationships among genes, proteins, chromatin accessibility peaks, and metabolites at the center of the analysis. Entity-level graphs can become prone to overfitting when the number of entities is small relative to the feature dimension, whereas feature-level graphs are harder to reuse when feature definitions are unstable across studies (as with ATAC-seq peak sets or platform-specific protein panels) or cross-platform alignment is weak. In practice, both views are often combined, with feature information guiding entity neighborhoods and entity neighborhoods in turn refining feature networks.

### Constructing graph structure

After nodes are chosen, the source of structure must be decided. Data-driven graphs are favored when discovery of cohort-specific patterns is desired, when sample sizes are adequate, and when batch effects can be controlled; they can reveal novel edges that curated resources lack. Knowledge-based graphs are preferred when interpretability and stability are paramount, when the data are noisy or scarce, or when the biology is well-charted (e.g. canonical pathways, PPIs, transcription factor-target maps). A hybrid strategy is often the most practical, with prior knowledge anchoring topology and semantics while the data adjust edge weights, remove spurious links, and add context-specific connections. Care is needed with purely data-driven graphs in the presence of strong confounders such as batch effects, library size, or compositional biases, and with purely knowledge-based graphs when priors are outdated, tissue agnostic, or uneven across omics. Governance also matters, and versioning of priors, inclusion of confidence scores, and periodic updates should be planned so that results remain reproducible.

### Specifying omics interactions

Attention then turns to how omics layers interact. An intra-omics graph is appropriate when signals mainly reside within a single layer, for example co-expression in transcriptomics, co-accessibility in chromatin, or structural neighborhoods in proteomics, or when each modality will be modeled separately before fusion. An inter-omics graph, often with multiple node types, is more suitable when cross-layer regulation is central to the hypothesis, such as transcription factors activating genes, miRNAs repressing mRNA, methylation modulating expression, or metabolites interacting with enzymes. Mixed designs are useful when both within-layer neighborhoods and cross-layer constraints carry information, and edge weights can be tuned to balance their contributions. Inter-omics graphs are better avoided when feature mapping across layers is unreliable, for example when it is unclear which regulatory regions or chromatin accessibility peaks should be linked to which genes, or when the two modalities share too few matched features to support stable cross-layer edges, or when the additional complexity overwhelms the available sample size. It is also important to consider edge direction and sign, such as activation or repression, wherever the underlying biology supports it, since this can sharpen both inference and interpretability.

### Integrating omics layers

With the graph semantics and omics interactions defined, the next step is to choose the integration stage. Early integration is appropriate when modalities are commensurate, well normalized, and rapid baseline models are needed; it offers simplicity but is sensitive to heterogeneity and missing data. Intermediate integration is preferred when each modality benefits from its own encoder because of differences in scale, sparsity, or noise characteristics, yet a joint representation is still needed to capture synergy. Attention, alignment, co-regularization, and cross-graph message passing are common strategies in this setting. Late integration is most suitable when modularity, interpretability, or robustness to missing modalities is critical, since each modality is modeled independently and the outputs are fused at the decision level using approaches such as ensembles, meta-learners, or consensus clustering. In practice, early fusion should be avoided for highly heterogeneous layers, late fusion should be avoided when cross-omics interactions carry essential signal, and intermediate fusion should be used cautiously when sample sizes are too small to support the added complexity.

### Adapting the model to practical constraints

At this stage, practical constraints further refine the final model choice. When sample size is small, it is better to favor simpler models or stronger regularization, and to use data-driven approaches with strong regularization or knowledge-based priors to reduce overfitting. When compute or memory is limited, scalable or sparse architectures are preferred, often leveraging sparse graphs, pooling, or lighter GNNs. When missing modalities are expected, modular or imputation-ready designs are advantageous, such as late fusion or partial-fusion intermediate designs that tolerate incomplete inputs. When interpretability is a major need, hybrid graphs anchored in curated priors, sparse edge structures, and attribution-ready models provide transparency and biological grounding. These constraints often interact with one another. Strong regularization aligns naturally with small sample settings, scalability pushes toward sparse architectures, modularity supports analyses with incomplete data, and interpretability is enhanced by hybrid designs or models that incorporate prior knowledge.

Table 2 highlights that AMOGEL [85] and IGCN [89] make observably different graph-construction choices even under the same multi-omics setting. These models show how the graph design becomes a chain of choices once the target is stated clearly. In IGCN, the primary target is patient-level classification (such as subtype prediction), so supervision attaches to patients and the most natural node schema is an entity-level graph in which each node is a patient. This choice makes data-driven similarity the simplest and most task-aligned edge semantics: IGCN builds one patient–patient graph per modality using cosine-similarity thresholding. As a result, the modeled relations are mainly within-layer, so the graphs are intra-omics by construction, while cross-omics complementarity is handled at the representation stage through intermediate integration, using per-omics GCN encoders followed by patient-specific attention (and additional regularization to reduce sensitivity to noisy similarity edges). AMOGEL begins with the same supervised objective of classification, but it explicitly adds a second goal of biomarker discovery and mechanistic interpretability, and that dual target changes the downstream decisions. To support feature-level attribution, AMOGEL adopts a feature-centric node schema so that learning and explanation operate directly over molecular entities rather than only over patient neighborhoods. This then motivates edges that are not defined by patient similarity but by relationships among features that are informative for the label: AMOGEL mines intra-omics and inter-omics associations to form a synthetic information graph and augments it with curated priors (such as PPI and pathway/ontology links), producing a hybrid structure with mixed relations. Because this unified multi-omics graph is constructed before message passing, it aligns with an early integration design.

In conclusion, design should be goal-first: node view follows the supervision target (entity-level for per-entity outcomes; feature-level for mechanisms), structure follows the need for discovery versus interpretability (data-driven, knowledge-based, or hybrid), interaction type follows the biological hypothesis (intra- versus inter-omics), and fusion stage follows the trade-off between simplicity, synergy, and modularity (early, intermediate, late). It is advisable to prototype at least two complementary pipelines, for example a hybrid graph with intermediate fusion and a modular late fusion ensemble, so that accuracy, stability, calibration, and interpretability together guide the final choice. Throughout, careful preprocessing, batch correction, and validation on external cohorts should be planned to ensure that the resulting graph model is both biologically credible and practically useful.

## Conclusion

Graph-based deep learning has quickly become a key approach for multi-omics integration because it aligns with the relational nature of biology, where genes, proteins, regulatory elements, cells and tissues act in networks rather than as isolated measurements. In this review, we organized the growing body of work along a few core design axes, including node schema, edge semantics, type of omics interaction, integration strategy, graph context, and model architecture, and used these to compare representative methods in bulk, single-cell and spatial settings.

As single-cell and spatial multi-omics technologies mature and become more accessible, we already see a shift in focus from bulk integration toward frameworks that integrate matched modalities at cellular and tissue resolution, often relying on richer and more complex graph constructions. Within spatial multi-omics, this trend is further reflected in methods that move beyond two-dimensional spot-neighborhood graphs toward frameworks that integrate molecular profiles with histology images, align representations across serial tissue slices, and reconstruct higher-order or three-dimensional tissue organization. These developments suggest that future spatial graph models will need to represent not only local tissue adjacency, but also morphology-aware, cross-slice, and multi-scale spatial relationships.

Viewing all of these methods within a shared design space makes it easier to recognize that many seemingly different pipelines are variations on common themes, which in turn helps with comparing models, identifying underexplored regions and tailoring approaches to specific biological or clinical questions. Across this landscape, the definition of the graph and its properties is often the most consequential design choice, since it encodes which entities are allowed to communicate, which relations are trusted and how information flows between features, cells and patients; this suggests that progress may come less from ever deeper neural stacks and more from graphs that better reflect relevant biology and are used thoughtfully for information propagation across layers and scales. Looking ahead, we expect further advances to depend on clearer and more realistic problem formulations, transparent and standardized benchmarks with explicit reporting of graph construction and prior knowledge, and tighter links between model predictions and experimental or clinical validation, so that graph-based multi-omics methods can evolve from proof-of-concept studies into robust tools for dissecting disease mechanisms and informing diagnosis and treatment.

**Key points**

- This review introduces a graph-centric review framework for multi-omics integration that organizes the field around graph design and usage rather than primarily around model architectures.
- We systematically compare graph-based multi-omics methods across bulk, single-cell, and spatial settings, emphasizing the strengths, limitations, and trade-offs of different graph design strategies.
- We outline a practical decision pipeline for graph-based multi-omics model development, spanning scientific objective definition, graph construction and curation, model training, and evaluation.

## Data Availability

This research does not involve the use of datasets. The analysis presented is qualitative in nature.
